# Exposome project for health and occupational research night shift cohort (EPHOR-NIGHT): a unique resource to advance research on night shift work and chronic disease

**DOI:** 10.1136/bmjopen-2025-106090

**Published:** 2025-12-05

**Authors:** Barbara N Harding, Gemma Castaño-Vinyals, Karin Broberg, Maria Albin, Caisa Laurell, Anne Helene Garde, Kirsten Nabe-Nielsen, Anne Emily Saunte Fiehn, Tara van der Grinten, Susan Peters, Roel C H Vermeulen, Manosij Gosh, Miranda Loh, Anjoeka Pronk, Manolis Kogevinas

**Affiliations:** 1 College of Population Health, The University of New Mexico, Albuquerque, New Mexico, USA; 2 Department of Non-Communicable Diseases and Environment, Instituto de Salud Global Barcelona, Barcelona, Catalunya, Spain; 3 Pompeu Fabra University, Barcelona, Spain; 4 CIBER Epidemiología y Salud Publica (CIBERESP), Madrid, Spain, Madrid, Community of Madrid, Spain; 5 IMIM (Hospital del Mar Medical Research Institute), Barcelona, Spain; 6 Institute of Environmental Medicine, Karolinska Institute, Stockholm, Sweden; 7 Department of Laboratory Medicine, Lund University, Lund, Skåne County, Sweden; 8 National Research Centre for the Working Environment, Copenhagen, Capital Region of Denmark, Denmark; 9 Department of Public Health, University of Copenhagen, Copenhagen, Capital Region of Denmark, Denmark; 10 Institute for Risk Assessment Sciences, Utrecht University, Utrecht, The Netherlands; 11 Environment and Health Unit, KU Leuven, Leuven, Flanders, Belgium; 12 Institute of Occupational Medicine, Edinburgh, Scotland, UK; 13 Netherlands Organization for Applied Research, TNO, The Hague, Netherlands

**Keywords:** Cardiovascular Disease, Cognition, EPIDEMIOLOGY, Health Workforce, MENTAL HEALTH, OCCUPATIONAL & INDUSTRIAL MEDICINE

## Abstract

**Abstract:**

**Purpose:**

The EPHOR-NIGHT cohort was established to investigate how night shift work influences biological pathways and chronic disease risk using a comprehensive working-life exposome approach, focusing on cardiometabolic, mental health, cognitive and biological ageing outcomes.

**Participant:**

The cohort includes 937 workers aged 20–65 years (88% female), primarily from the healthcare sector (96%) in Spain, Sweden, Denmark and the Netherlands. Participants were categorised as permanent day (39%), permanent night (35%) or rotating/other shift workers (26%). Data collection included questionnaires, daily ecological momentary assessments, wearable sensors tracking light, physical activity, heart rate and environmental exposures and biological samples (blood collected once and saliva collected during five points across the day), with harmonised protocols across countries.

**Findings to date:**

From the 937 participants contributing data to the cohort, 708 had complete information from questionnaires, sensors and blood and saliva, with subsets undergoing advanced biological analyses, including genomics, targeted and genome-wide DNA methylation, telomere length and mtDNA copy number, metabolomics, transcriptomics, proteomics, hormone profiling and inflammatory biomarkers and blood metals. Many reported prevalent chronic conditions, including anxiety (27%), depression (18%) and metabolic disturbances. Night shift and rotating shift workers had greater exposure to long shifts and more scheduled rest days compared with day workers. Sleep duration and quality were poorest among permanent night shift workers.

**Future plans:**

A 2-year follow-up was completed in June 2025, including the collection of additional biomarker data, psychosocial work environment data and data related to female sexual and reproductive health. Findings from the EPHOR-NIGHT study aim to inform prevention strategies and occupational health policies. Data will be made available to support broader research efforts on shift work and health.

**STRENGTHS AND LIMITATIONS OF THIS STUDY**

- The EPHOR-NIGHT cohort is among the most comprehensive exposome-based investigations of night shift work and health to date.
- The data collected enable in-depth investigation of multiple biological pathways relevant to several non-communicable diseases.
- The data include advanced omics analyses and objective measures of sleep and light exposure, supporting mechanistic research on circadian disruption and chronic disease.
- Most current analyses are cross-sectional, limiting causal inference.
- The study consists predominantly of healthcare workers, limiting generalisability to other occupational groups.

## Introduction

 Night shift work is widespread across various industries. Globally, millions of individuals engage in night shift work, with varying prevalence by region and occupation; in Europe, it is estimated that 19% of the population engages in night shift work.^1^ Night shift work is essential for maintaining 24-hour operations in sectors such as healthcare, transportation and manufacturing, and its prevalence is increasing due to expanding human activities over the 24-hour day. Night shift work misaligns individuals’ biological rhythms with their external environment, leading to circadian disruption. This misalignment interferes with the regulation of sleep-wake cycles, hormone secretion and metabolic processes, and countless other biological systems, often resulting in chronic sleep deprivation and reduced physiological recovery.^2^

Experimental and epidemiological evidence shows that long-term disruption of endogenous circadian rhythms, in particular due to exposure to light during the biological night, may be associated with a wide range of common non-communicable diseases (NCDs), including cancers,^3^,^5^ cardiovascular diseases,^6^ major metabolic disorders (obesity and type 2 diabetes)^7 8^ and mental health disorders.^9^ Night shift work is considered a probable human carcinogen by the International Agency for Research on Cancer (IARC),^10^ highlighting its potential link to cancer development. The 2019 IARC evaluation stressed the lack of information on mechanisms and the particular need for population-based mechanistic data to understand how night shift work impacts health.^11^ Such knowledge is essential to minimise health risks involved with shift work in situations where shift work cannot be avoided. Despite mounting evidence on the health risks associated with night shift work, significant gaps remain in understanding the interplay of potential risk factors responsible for these health effects, namely, occupational exposures, lifestyle factors, the environment and individual susceptibility.

**Figure 1:**
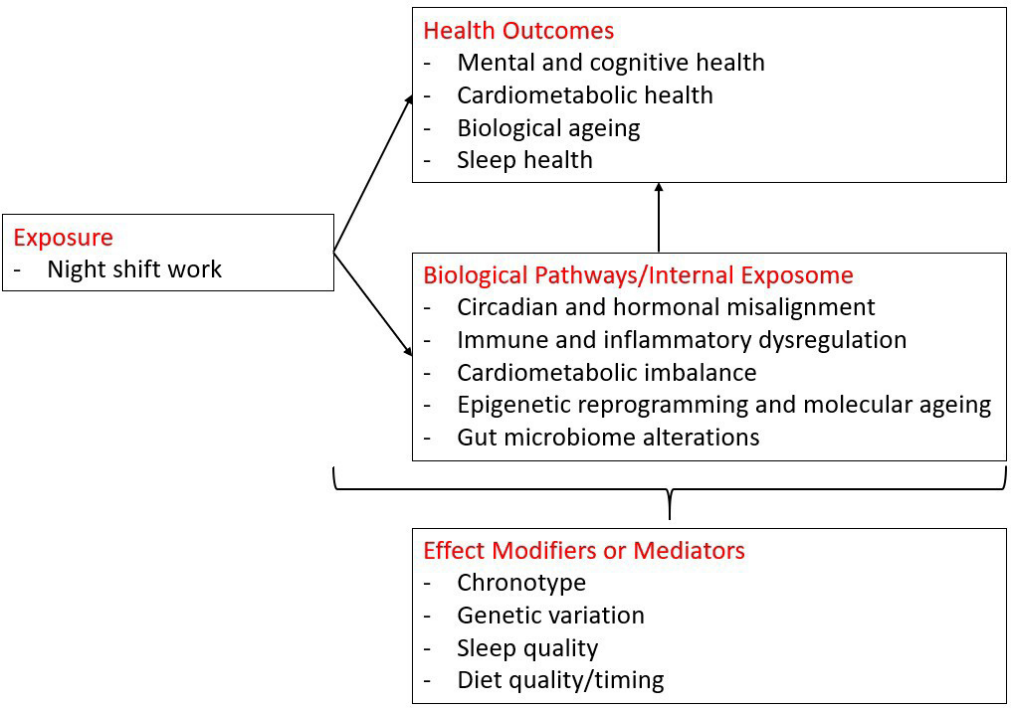
Conceptual framework of the EPHOR-NIGHT cohort showing the exposome approach to studying night shift work and health. Occupational, behavioural and environmental exposures lead to circadian, metabolic, immune and molecular changes that are associated with mental, cognitive, cardiometabolic and biological ageing outcomes, modulated by chronotype, genetic variation, sex and sleep quality.

The complexity of relevant exposures that influence disease risk among night shift workers calls for further investigation within an exposome framework.^12 13^ There is a need for more comprehensive insights into exposure metrics and biological pathways, which consider both occupational and non-occupational risk factors and genetic make-up. By leveraging advanced exposomics methodologies,^14 15^ the EPHOR-NIGHT cohort was established to examine how the working-life exposome among night shift workers affects key body functions and biological pathways, and examine these mechanisms in relation to the development of (1) cardiometabolic outcomes, (2) mental health outcomes, (3) cognition and (4) biological ageing. We hypothesised that night shift work would be associated with perturbations in a variety of biological pathways, and that workers engaged in night shift work would have a greater prevalence of negative cardiovascular, mental health, cognitive health, as well as ageing-related outcomes when compared with day shift workers. We also hypothesised that shift work may influence many lifestyle habits including sleep characteristics and timing, physical activity, diet quality and timing, which in turn influence disease outcomes. A conceptual framework was created (figure 1) to overview the key exposures, outcomes and mechanisms of interest. Using an exposome approach, the EPHOR-NIGHT cohort was designed to test these hypotheses and to account for the mixture of exposures that together contribute to differences in health among day and night shift workers. This research has the potential to identify actionable strategies to mitigate health risks in this large, high-risk population.

In this paper, we overview the creation of the EPHOR-NIGHT cohort, summarising the data collected so far, overviewing aspects of the newly created cohort and sharing future directions.

## Cohort description

The EPHOR-NIGHT cohort enrolled 937 workers including 366 (39%) permanent day shift workers, 325 (35%) permanent night shift workers and 246 (26%) workers in rotating or alternative shift schedules between the ages of 20–65 years from four centres: (1) Barcelona, Spain, (2) Stockholm, Sweden, (3) several areas in Denmark and (4) Utrecht, The Netherlands.

### Data collection

Due to feasibility constraints related to COVID-19 regulations, which varied by country and affected the ability to collect certain types of data, a ‘core’ data collection protocol was established (table 1). Deviations from the core protocol sometimes occurred, and these are indicated below. Additional data collection methods and analyses were conducted in subsets of the population. All respondents completed a baseline questionnaire and underwent five continuous days of monitoring. During the 5 days of data collection, a majority of participants responded daily to an ecological momentary assessment phone application, had anthropometric measurements taken in person and gave saliva samples and blood samples. A large subset of participants also completed a series of cognitive examinations and wore several sensors. Table 2 provides an overview of how many participants had certain types of key data collected, and online supplemental appendix 1 provides a visual overview of the different aspects of data collection, and when they occurred throughout the 5 days of continuous data collection.

**Table 1:** Overview of the types of data collected, and which collection activities were considered ‘core’ versus conducted in a subsample only

Variables/samples	Core	Variables/samples	Core
**Baseline questionnaire info:**		**In person height, weight, BP**	
Medication use	x	Height-in person	
Medical history	x	Weight-in person	
Alcohol use	x	Waist to hip ratio-in person	
Smoking	x	Blood pressure-in person*	x
Physical activity	x	**Cognitive**	
Sleep	x	Mental/cognitive	x
Chronotype	x	**Biologic samples**	
Diet and meal timing	x	Venus blood samples	x
Working time, duration of night work	x	Dried blood spot	
Psychosocial	x	Saliva	x
Height and weight (self-report)	x	**Variables generated from analyses of biologic samples**	
Residence		Metabolic syndrome*	x
**Ecological momentary app**	x	Diabetes†	x
**Sensor-collected** [Table-fn T1_FN2] †		Allostatic load‡	
Fine dust (PM1, PM2.5, PM10)		Telomere length, mtDNA copy number	
Temperature		Hormones	x
Relative humidity		Cytokines/chemokines	x
Noise		C-reactive protein	
Light	x	Targeted DNA methylation	
UV		Omics—genomics, epigenomics, transcriptomics and proteomics	
Activity from accelerometer		Appetite hormones leptin and ghrelin	
Heart rate		Microbiome	
Chemical exposures (passive sampler)			

* Measured using HbA1c, triglycerides, cholesterol, blood pressure, waist circumference/BMI.

† Measured using HbA1c.

‡ Measured using hormone and inflammation biomarkers, lipids, blood pressure and heart rate.

BMI, body mass index; BP, blood pressure.

**Table 2:** Overview of data types available within the EPHOR-NIGHT dataset, stratified by centre

Centre	With blood, saliva, sensors, baseline Q (n)	With blood, sensors, baseline Q (n)	With hormones (n)	With telomere length and mtDNAcn (n)	With metabolomics (n)	With immune markers (n)	With GWAS, EWAS (n)	With full omics—GWAS, EWAS, metabolomics, proteomics (n)
Spain	368	368	400	400	404	400	200	100
Sweden	165	167	167	171	168	150	109	100
Denmark	175	219	233	197	200	149	100	100
The Netherlands*	0	0	0	50	100	50	0	0
**Total**	**708**	**754**	**800**	**818**	**872**	**749**	**409**	**300**

* The samples indicated for The Netherlands are from the Klokwerk study.

Baseline Q, baseline questionnaire; EWAS, epigenome-wide association study; GWAS, genome-wide association study; mtDNAcn, mitochondrial DNA count number.

### Inclusion and exclusion criteria; exposure definition

According to the protocol, night shift workers were required to be working a minimum of two consecutive night shifts per week, and to have worked night shifts for 3+ years prior to enrolment to be considered exposed. A night shift was defined as a working schedule that involved working 4+ hours of the shift between 00:00 hours and 06:00 hours, a definition modified from the International Agency for Cancer’s consensus definition for night shift work.^16^ For day shift workers, participants were not allowed to be currently working night shifts, nor have worked night shifts for the prior 3 years at the time of enrolment. Incidental night shift work was allowed (working less than one night shift per month, however, ≥1 week must have elapsed after an incidental night shift before the day shift worker was enrolled into the study). A day shift was defined as a working schedule that involved working 4+ hours of the shift between 07:00 hours and 18:00 hours (shift must start at 06:00 hours or afterward).

### Study population

Data for this cohort study were collected between February 2022 and June 2024 in four centres: Barcelona, Spain (bus workers and healthcare workers), Stockholm, Sweden (healthcare workers), several areas in Denmark (healthcare workers) and Utrecht, The Netherlands (healthcare workers).

In Spain, data were collected between February 2022 and July 2023. Participants were recruited from a bus company (Transports Metropolitans de Barcelona) and hospitals (Hospital Clinic and several hospitals within the Parc Salut Mar hospital group) via flyers and intranet announcements by their labour health departments. After eligibility screening, 408 participants enrolled, with 403 (34 bus company, 369 hospitals) completing the study. Hospital participants were primarily nurses, while the bus company included drivers and administrative staff, who worked varying shift schedules. At Hospital Clinic, day shifts ran from 8:00 to 15:00, while night shifts followed a 2-week cycle: five nights (22:00–08:00) in the long week and two in the short week. At Parc Salut Mar, day shifts were 7:30–15:00, afternoon shifts were from 14:30 to 21:45 and night shifts followed a similar 2-week cycle: four nights in the long week and three in the short week (21:00–8:00). Data collection followed the full study protocol, including in-person visits, biological sampling and sensor use.

In Sweden, data were collected between June 2022 and June 2024. Participants were recruited mainly from the healthcare sector in the Stockholm region, through human resources and nurse managers at different departments. Recruitment was supported with email communications and in-person presentations at clinics. Participants who showed interest were contacted to evaluate inclusion criteria and working schedule. Healthcare staff who met the inclusion criteria and had no limitations (hygienic and technical regulations) to wear sensors at the workplace were included. After eligibility screening, 212 participants were enrolled and 170 completed the study. Most participants were nurses or assistant nurses, and 14 participants were from non-healthcare occupations (eg, postal workers, police). Participants typically worked permanent dayshift (around 7:00 to 15:30) or permanent night shift (around 21:00 to 7:00) with fewer participants with a rotating schedule (11%). Participants underwent in-person assessments and followed the main data collection protocol.

In Denmark, data were collected between September 2022 and April 2024 in all of the five regions of Denmark as part of the 1001 nights cohort.^17^ Participants were recruited from hospitals with help from hospital management, often in collaboration with employers’ and employees’ representatives. After eligibility screening, 322 enrolled, and 294 completed the study. All participants were women working in healthcare, mainly nurses, nursing assistants, midwives, medical doctors, biomedical laboratory scientists and administrative staff employed in hospital departments including somatic or psychiatric care, for example, medical and surgical wards or emergency or outpatient departments. Typical day shifts ran from 07:00 to 15:00, evening shifts from 15:00 to 23:00, and night shifts from 23:00 to 07:00. Data collection was conducted in person and by participants themselves, and participants generally adhered to the protocol regarding biological and sensor-based measurements but did not complete cognitive testing.

In the Netherlands, data were collected between May 2023 and January 2024. Participants were recruited from the Nightingale cohort of female registered nurses.^18^ Nightingale participants were contacted through a mailing list with an invite to participate and recruited if they were officially registered as a nurse in the national BIG register (Beroepen in de Individuele Gezondheidszorg), the official Dutch registry for healthcare professionals, including nurses. After eligibility screening, 93 enrolled, with 70 participants who completed the baseline questionnaire ultimately included in the final cohort. Typical day shifts ran from 07:00 to 15:00, evening shifts from 15:00 to 23:00, and night shifts from 23:00 to 07:00. The majority of workers worked three night shifts in a row, but an average of one night shift per week. Unlike other centres, data collection was conducted remotely: participants completed online questionnaires, cognitive testing via video call and the biological samples and light sensor (HOBO) were sent via mail.

Participants from the Klokwerk study (n=50) were included in selected analyses of EPHOR-NIGHT, for example, cytokine analysis, using the same laboratory procedures.^19^ The Klokwerk study included Dutch female nurses and paramedic staff aged 18–67 years who had recently started working in night shifts (within the last 2 years), had long-term experience of working in night shifts (over 5 years), or had not worked in night shifts in the last 5 years before recruitment (defined as ‘day-workers’). To participate in the study, subjects had to agree to blood sampling and filling out the questionnaires.

### Ethics

In three of the centres, Spain (CEIm-Parc de Salut Mar approval #2021/9845), Sweden (Stockholm Ethics Review Authority approval #2021-02386) and Denmark (Danish National Committee on Health Research Ethics approval #H-21077744), the protocol underwent review by the relevant ethics committee and received approval. In the Netherlands, the study underwent review by the Medical Research Ethics Committee (MREC) review board and was deemed exempt. At each of the participating centres, participants were given information sheets providing details about the study, study objectives and data collection protocol. Prior to enrolment, all participants signed informed consent forms.

### Data collected

#### Baseline questionnaire

Subjects completed a baseline questionnaire (online supplemental appendix 2) to provide information on lifestyle, medical history, sleep (with several questions, including the Pittsburgh Sleep Quality Index (PSQI)),^20^ diet, working conditions, night-shift duration and psychosocial factors. Chronotype was assessed using the Morningness-Eveningness Questionnaire (MEQ).^21^ The Dutch participants filled in a slightly abbreviated questionnaire based on the questionnaire used in the Nightingale Study (an English translation of this questionnaire is available in online supplemental appendix 3). The Danish participants completed a slightly modified version as well (online supplemental appendix 4).

#### Ecological momentary assessment

Participants used the ‘How am I’ smartphone application to report daily on commuting, sleep, diet, lifestyle and psychosocial factors. The dietary questions were asked two times per day, and the other questions were asked once daily (online supplemental appendix 5). The Danish participants used a slightly modified local questionnaire that had overlapping questions (online supplemental appendix 6).

#### Anthropometric measurements (in-person visit)

During an in-person visit, height, weight, waist and hip circumferences, and seated blood pressure were recorded. Height and weight were measured with a stadiometer, while waist and hip circumferences were taken using a tape measure at standardised anatomical landmarks. Blood pressure was assessed at room temperature using an automated device, with three readings taken and the average calculated from the second and third measurements.

**Cognitive examinations**

Cognitive function was evaluated using both cold (non-emotional) and hot (emotional) cognition tests. Cold cognition tests were conducted via video call on a day off from work under standardised conditions, while hot cognitive tests were conducted on a day of work following the shift. The tests included:

Digit Span (Wechsler Adult Intelligence Scale – IV; two subtasks of the Digit Span, which is part of the Wechsler Adult Intelligence Scale —Fourth Edition^22^: Participants repeated a sequence of numbers either in the same order (forward) or in reverse (backward). The forward task assessed short-term auditory memory, while the backward task measured working memory.^23^

Semantic Fluency: Participants named as many animal species as possible in 1 min,^24^ assessing language production, cognitive abilities and non-motor processing speed.^25^

Controlled Oral Word Association Test^26^: Participants listed words beginning with a specific letter within 1 min across three trials. The letters varied by country based on validated adaptations. This test assessed phonemic fluency and cognitive abilities via motor processing speed.^25^

Hot cognition (ie, emotional processing and reasoning) was assessed with the Dot Probe Task, measuring attentional bias to emotional stimuli.^27^ Conducted on a workday after the shift via smartphone, tablet or computer (∼8 min), participants viewed paired images (neutral vs neutral/threatening) before identifying the location of a dot appearing in place of one image.^28^

#### Sensors

Several wearable sensors were used to collect environmental and physiological data. All sensors were worn during the 5 days of data collection, unless otherwise specified below. Information on how many participants utilised the sensors is included below and in table 2.

1. HOBO Light Intensity Logger (Onset HOBO Data Logger, UA-002–64, Massachusetts, USA): A small pendant recorded ambient light exposure every 15 s. Worn at chest level during the day, it was placed on a bedside table during sleep (n=807 had HOBO data).
2. Kronowise Wrist Device (Kronowise 3.0, Kronohealth, Murcia, Spain)^29^: Worn continuously for 5 days by a subset of participants in Spain and Sweden, it measured light exposure in three spectral bands (visible, blue light of 460–490 nm, and infrared >800 nm), activity, sleep and skin temperature (n=562 had Kronowise data).
3. Heart Rate Monitor (Polar H10, Kempele, Finland): A Polar heart rate monitor was used by a subset of participants in Spain and Sweden to obtain heart rate variability data. Participants wore it during waking hours, synchronised data using an app specially designed for the study, ‘EphorPolar’, and downloaded data before removing it at night (n=336 had Polar data).

In subsets, we additionally used the following sensors: Sensor Box (n=102): Developed within the EPHOR-NIGHT cohort, this sensor measured ambient temperature, humidity, sound, light intensity, UV intensity and particulate matter (PM1, PM2.5, PM10). The sensor was worn on a belt during waking hours and removed for charging and data download during sleep. Passive Samplers (worn during 1 day) (n=27): a Tenax-PDMS combined sampler which operates passively and is analysed for a wide screening analysis of over 2000 volatile organic compounds (VOCs) and a smaller set of specific semi-volatile organic compounds.^30^ These small samplers were worn at chest or shoulder height during the day and placed in the bedroom or bathroom during sleep or showering.

#### Biological samples

Blood samples (38.5 mL) were collected for each participant between 6:00-10:00h to ensure homogeneity for all shifts. In the instances (particularly in Denmark) where this was not possible, the time of collection was indicated. Saliva samples were collected at five time points daily, including upon waking, 45 min after waking, 4 and 10 hours post-waking, and before bed. Dried blood spots were also collected in a subset of participants. Saliva and blood samples were collected to analyse biomarkers, ensuring participants had worked at least two consecutive night shifts (1 night in Denmark) before sampling. In this way, we aimed to collect biomarker data which reflected changes expected based on the shift exposure, and we avoided collecting biomarkers from participants who had just had one or more free days from work. Key analyses included epigenome- and genome-wide data (EWAS and GWAS), targeted DNA methylation, telomere length, mtDNA copy number, transcriptomics, metabolomics, proteomics, hormones (including melatonin), inflammatory biomarkers, metals in whole blood, appetite hormones leptin and ghrelin and the gut microbiome. More information on these analyses and the methods is available in table 3.

**Table 3:** Overview of biological analyses

Biological matrix	Endpoint measured	Method(s) used
Saliva	melatonin, cortisol, cortisone, testosterone, androstenedione and related metabolites	UHPLC-MS/MS^32 33^
Saliva		
EDTA plasma	Cytokines/chemokines (**Cytokines**: G-CSF, GM-CSF, IFN-α, IFN-γ, IL-1β, IL-1RA, IL-2, IL-2R, IL-4, IL-5, IL-6, IL-7, IL-8, IL-10, IL-12 (p40/p70), IL-13, IL-15, IL-17, TNF-α **Chemokines**: Eotaxin, IP-10, MCP-1, MIG, MIP-1α, MIP-1β, RANTES **Growth factors**: EGF, FGF, HGF, VEGF)	Cytokine Human Magnetic 30-Plex Panel
EDTA plasma	Metabolic biomarkers	NMR^34 35^
EDTA plasma	Proteomics	Sample prepared using the iST-BCT kit, and analysed using the evoSEP-ZenoTOF platform
DNA isolated from EDTA whole blood	Genetic markers for GWAS analysis	Infinium Global Screening Array-24 v3.0 BeadChip
DNA isolated from EDTA whole blood	DNA methylation analysis for EWAS analysis	Infinium MethylationEPIC v2.0 kit
DNA isolated from EDTA whole blood	DNA methylation analysis-sequence specific	Pyrosequencing (Qiagen Q24/Q48)- methylation analysis
DNA isolated from EDTA whole blood	Telomere length	qPCR^36^
DNA isolated from EDTA whole blood	Mitochondrial copy number	qPCR^36^
Whole blood (PAX RNA tube)	mRNA expression	NanoString technology
Whole blood	Metals (lead, cadmium, etc)	ICP-MS (Agilent 7900)^37 38^
EDTA plasma	Leptin and ghrelin	ELISA
Stool	Gut microbiome relative abundance and diversity	16S rRNA

EGF, epidermal growth factor; EWAS, epigenome-wide association study; FGF, fibroblast growth factor ; G-CSF, granulocyte colony-stimulating factor; GM-CSF, granulocyte-macrophage colony-stimulating factor; GWS, genome-wide association study; HGF, hepatocyte growth factor; IFN-γ, interferon-gamma; NMR, nuclear magnetic resonance ; qPCR, Quantitative PCR; TNF-α, interferon-alpha; UHPLC-MS/MS, Ultra-High Performance Liquid Chromatography-Tandem Mass Spectrometry; VEGF, vascular endothelial growth factor.

### Follow-up data collection

In addition to the baseline data collection, a 2-year follow-up study was completed for 428 participants. The follow-up spanned July 2024–June 2025. Only participants at the Spanish and Swedish centres were invited to participate. In the Spanish centre, of 403 participants at baseline, 7 did not give permission to re-contact them in the future. Out of the 396 that were contacted to participate in the follow-up, 328 participated (response rate 83%). In the Swedish centre, out of 170 who were contacted to participate in the follow-up, 109 participated (response rate 64%). The follow-up data collection included a questionnaire (this time adding questions about psychosocial work environment including 22 questions from the Copenhagen Psychosocial Questionnaire^31^ and female sexual and reproductive health questions), a sleep diary used during 5 days, the use of the Kronowise light and actigraphy monitor^29^ during 5 days, the collection of anthropometric measurements and venous blood in-person on a working day and the collection of stool samples for a subset of the population (n=206).

### Data harmonisation

We conducted extensive harmonisation efforts to identify differences in logbook and questionnaire data stemming from protocol variations, language or question wording. In cases of significant differences, centre-specific datasets were retained alongside the common dataset for potential future use. Data collection in Spain, Sweden and Denmark showed the greatest alignment, allowing for comprehensive data availability across most variables within these three subsets. In addition, because of different shift schedules in each of the centres, we planned the protocol to synchronise the clock hour of biological sample collection, thereby reducing the influence of normal circadian rhythm differences.

### Public and patient involvement

During the design of the study, we consulted with occupational health groups and representatives in multiple centres and integrated their feedback on feasibility and acceptability into the protocol and recruitment procedure. In several centres, pilot studies were conducted to test the feasibility of the protocol. We also involved study participants in multiple online sessions to give feedback on early findings from the research and to hear their feedback on the study.

## Findings to date

In this paper, we summarise how many participants completed various aspects of the study protocol, and for how many we have biological samples for different biomarker analyses. We conducted a descriptive overview of demographic, lifestyle and night shift-related characteristics of the participants in the study. We also stratified by centre to describe centre-specific characteristics. For categorical variables, we provide the number and percentage and for continuous variables, we provide the mean and standard deviation. Finally, we provide more detailed information on shift exposure, sleep and other work-related characteristics of our population. These variables are described according to shift schedules: permanent day shift workers, permanent night shift workers and rotating shift workers. All analyses were conducted in STATA version 18 and R Studio V.2024.9.0.375.

In total, 937 workers participated in the EPHOR-NIGHT cohort. Of these, 708 completed the full data collection protocol, with data available on blood, saliva, sensors and questionnaires. In addition, 800 participants had saliva analysed for hormones, 749 had serum samples analysed for immunological biomarkers and 300 had complete omics data in blood (GWAS, EWAS, metabolomics and proteomics) (table 2). The vast majority of the cohort was of European origin (89%) (table 4).

**Table 4:** Demographic characteristics of the EPHOR-NIGHT sample, stratified by centre (N=937)*

	Overall	Spain	Sweden	Denmark	The Netherlands
Characteristics	n=937	n=403	n=170	n=294	n=70
Age (mean (SD))	43.93 (12.42)	42.87 (13.26)	46.70 (10.80)	42.03 (11.85)	51.42 (9.35)
Female	823 (87.6)	307 (76.0)	151 (88.8)	295 (100.0)	70 (100.0)
European origin	768 (88.5)	353 (87.6)	132 (77.6)	283 (95.9)	–
Education					
Secondary studies or less	295 (31.4)	178 (44.1)	45 (26.5)	53 (18.0)	19 (27.1)
University or more	644 (68.6)	226 (55.9)	125 (73.5)	242 (82.0)	51 (72.9)
Current smokers	194 (20.7)	105 (26.0)	38 (22.4)	49 (16.7)	2 (2.9)
Alcohol use					
Former	39 (6.1)	16 (4.0)	6 (3.5)	–	17 (24.3)
Never	157 (24.4)	140 (34.7)	17 (10.0)	–	0 (0.0)
Current	448 (69.6)	248 (61.4)	147 (86.5)	–	53 (75.7)
Marital status					
Married or living as a couple	598 (63.8)	219 (54.2)	111 (65.3)	218 (74.1)	51 (72.9)
In relationship but living alone	80 (8.5)	36 (8.9)	12 (7.1)	27 (9.2)	5 (7.1)
Single	173 (18.5)	98 (24.3)	33 (19.4)	36 (12.2)	6 (8.6)
Other	86 (9.2)	50 (12.6)	14 (8.3)	13 (4.4)	8 (11.4)
Night shift worker	564 (60.1)	211 (52.2)	85 (50.0)	206 (69.8)	62 (88.6)
Have previously worked night shifts	678 (78.7)	310 (76.7)	66 (64.7)	233 (81.2)	69 (100.0)
Existing conditions†					
Cardiac arrhythmia	53 (5.7)	22 (5.4)	14 (8.3)	9 (3.1)	8 (11.8)
High cholesterol/triglycerides	167 (17.9)	114 (28.3)	7 (4.2)	38 (12.9)	8 (11.9)
Hypertension	109 (11.7)	45 (11.2)	29 (17.2)	28 (9.5)	7 (10.1)
Asthma	119 (12.8)	51 (12.7)	32 (19.0)	35 (11.9)	1 (1.5)
Depression	169 (18.1)	75 (18.6)	46 (27.4)	43 (14.6)	5 (7.4)
Anxiety	235 (27.2)	150 (37.2)	52 (31.0)	33 (11.2)	–

Missing values: % are calculated from non-missing values. Data were missing for alcohol use 31%, all other variables <1%.

‘–’ empty cells indicate that these questions were not asked or relevant.

* n (%) for categorical variables and mean (SD) for continuous variables.

† In Spain, Sweden and Denmark this question was worded, ‘Has your doctor ever told you that you have any of the following diseases…?’ While in The Netherlands, it was worded, ‘Has your doctor told you after 1 January 2017 that you have…?’.

Of the full study population, 60% worked schedules including night shifts (of these, 58% were permanent night and 42% were rotating night shift workers) and 88% were women. The cohort included participants from Spain (43%), Sweden (18%), Denmark (31%) and the Netherlands (8%). The cohort was predominantly healthcare workers (96%) who had completed university studies (69%). The cohort included 6% with an existing cardiac arrhythmia, 18% with high cholesterol or triglycerides, 12% with hypertension, 13% with asthma, 18% with depression and nearly a third with anxiety (27%) (table 4). In online supplemental appendix 7, we further stratify the Spanish sample into transportation workers (n=33) and healthcare workers (n=370) to describe demographic differences between workers in each of these sectors, finding that the transportation workers were more often men, more likely to have completed secondary studies or less, more likely to be married or living as a couple, more likely to have previously worked night shifts, reported lower levels of smoking but higher levels of current alcohol use and had a different distribution of existing conditions than those in the healthcare sector.

Out of the cohort, 39% worked permanent day shifts, 35% worked permanent night shifts, 25% worked rotating shifts with nights, with 1% working permanent evenings or rotating shifts without nights (table 5). On average within the sample, workers had engaged in almost 11 prior years of work involving night shifts, with the greatest duration of prior night shift work among the rotating shift workers (12 years) and then permanent night shift workers (10 years). Permanent night workers had a worse sleep quality index score (9.5 compared to 8.2 among permanent day shift workers and 8.7 among rotating shift workers). Permanent night shift workers also reported shorter sleep duration (9% with <5 hours compared to 5% in the overall sample). Permanent night shift workers and rotating night shift workers also had the greatest frequency of long shifts (defined as 12 or more hours at a time), with 11% of permanent night shift workers, 9% for rotating shift workers and only 4% of permanent day shift workers reporting they worked multiple long shifts/week or more. Permanent night shift workers and rotating shift workers had the most scheduled days off during the week, with 70% of night shift workers and 48% of rotating shift workers, respectively, reporting 3 or more days of rest per week on average, compared to only 10% of permanent day shift workers who reported 3+ days of rest per week.

**Table 5:** Additional information on sleep and shift-specific characteristics of the EPHOR-NIGHT cohort (N=937)*

	Overall (N=937)	Permanent day (n=366)	Permanent night (n=325)	Rotating/other (n=246)
Female	822 (87.7)	315 (86.1)	263 (80.9)	244 (99.2)
Physical activity (METs) (mean (SD))	2017.4 (2108.6)	2168.6 (2077.3)	2285.7 (2482.9)	1442.7 (1402.0)
PSQI score (mean (SD))	8.8 (2.8)	8.2 (2.6)	9.5 (3.2)	8.7 (2.4)
Sleep duration (hours)				
>7	152 (17.6)	67 (18.7)	53 (16.7)	32 (17.2)
6–7	468 (54.3)	212 (59.2)	133 (41.8)	123 (66.1)
5–6	199 (23.1)	69 (19.3)	104 (32.7)	26 (14.0)
<5	43 (5.0)	10 (2.8)	28 (8.8)	5 (2.7)
Years of night shift work (mean (SD))	10.0 (10.8)	2.6 (5.2)	14.4 (10.0)	14.0 (12.1)
Frequency of long shifts				
never	575 (61.4)	232 (63.6)	244 (75.1)	99 (40.2)
**≤**1x/month	200 (21.4)	84 (23.0)	34 (10.5)	82 (33.3)
Every other week	56 (6.0)	14 (3.8)	10 (3.1)	32 (13.0)
1 x/week	33 (3.5)	20 (5.5)	3 (0.9)	10 (4.1)
Multiple shifts/week	56 (6.0)	13 (3.6)	23 (7.1)	20 (8.1)
Always	16 (1.7)	2 (0.5)	11 (3.4)	3 (1.2)
Night shifts per week				
1 /week	91 (12.5)	0 (0.0)	0 (0.0)	91 (40.1)
2 /week	106 (14.6)	0 (0.0)	21 (6.5)	85 (37.4)
3 /week	134 (18.5)	0 (0.0)	101 (31.2)	33 (14.5)
4 /week	165 (22.7)	0 (0.0)	153 (47.2)	12 (5.3)
5+/week	53 (7.3)	0 (0.0)	49 (15.1)	4 (1.8)
Self-roster ability (yes)	449 (47.9)	177 (48.4)	100 (30.8)	172 (69.9)
Days off/week				
0	26 (2.8)	24 (6.6)	1 (0.3)	1 (0.4)
1	63 (6.7)	38 (10.4)	8 (2.5)	17 (6.9)
2	467 (49.9)	267 (73.0)	87 (26.9)	113 (45.9)
3	316 (33.8)	32 (8.7)	186 (57.4)	98 (39.8)
**≥**4	64 (6.8)	5 (1.4)	42 (13.0)	17 (6.9)
Melatonin use	162 (17.3)	19 (5.2)	56 (17.3)	87 (35.5)

Missing values: percentages are calculated from non-missing values. Data were missing for 3% years of night shift work, 8% sleep duration, 12% sleep quality score and all others <2% missing.

“-“ empty cells indicate that these questions were not asked or relevant.

* n (%) for categorical variables and mean (SD) for continuous variables.

MET, metabolic equivalent of task.

### Strengths and limitations

The EPHOR-NIGHT cohort is among the most comprehensive exposome-based investigations of night shift work and health to date, including 937 participants from four European countries. A key strength is the application of a harmonised, multisite protocol combining detailed questionnaire data, ecological momentary assessments, wearable sensors and extensive biological sampling. This enables in-depth investigation of multiple biological pathways relevant to several non-communicable diseases including cardiometabolic, mental health, cognitive and ageing-related outcomes in real-life working conditions.

The study applies an exposome framework to characterise the complex mixture of occupational, behavioural and environmental exposures in night shift workers. It also integrates advanced omics analyses (genomics, epigenomics, transcriptomics, metabolomics, proteomics) and objective measures of sleep and light exposure, supporting mechanistic research on circadian disruption and chronic disease.

However, several limitations should be considered. Most current analyses are cross-sectional, limiting causal inference. Nevertheless, a prospective component is already underway, with 2-year follow-up data collection completed in multiple centres. This will allow assessment of temporal changes in exposures and health outcomes. While the inclusion of multiple countries enhances generalisability, the predominance of healthcare workers (96%) may limit applicability to other occupational groups. While the EPHOR-NIGHT study represents one of the largest and most integrative mechanistic investigations of night shift work in human populations to date, which will allow for the identification of specific biological pathways underlying circadian disruption, the cohort size may limit statistical power for highly multifactorial or multi-omics analyses. Finally, logistical and contextual differences across sites, including remote versus in-person protocols and COVID-19-related constraints, introduced some variation in data completeness across centres.

## Future plans

This study will advance our understanding of the biological pathways linking night shift work to health outcomes using an exposome approach. By providing new evidence on cardiometabolic health, mental health, cognition and biological ageing, it will contribute to the development of policies aimed at mitigating night shift-related health risks. Extended follow-up with repeated measurements will enhance the prospective evaluation of these pathways and effects. Already, a 2-year follow-up has been completed for the Spanish and Swedish centre populations. We plan to conduct longitudinal analyses examining associations with repeated measures of biomarkers (eg, omics and immune analyses). We also plan to explore psychosocial work environment factors and female sexual and reproductive health outcomes. Additionally, the study will enable the integration of advanced biological analyses, such as microbiome and epigenetic profiling, and foster international collaborations to maximise scientific insights from the data. Furthermore, there is a microbiome study underway with microbiome data collected at the baseline and follow-up visits for the Spanish cohort. In Sweden, ongoing analysis of adipose tissue epigenomics and lipidomics will elucidate key changes in a target tissue for circadian disruption. To further support research, data will be made openly available to the scientific community, promoting transparency and broader exploration of findings.

From a prevention and regulatory perspective, our findings are directly relevant to the European Directive 2003/88/EC, which defines night work as employment involving ≥3 of the working day between 00:00 and 05:00, a definition that is closely aligned with our night shift definition of any working schedule involving ≥4 hours between 00:00 and 06:00. While the Directive provides the legal framework for regulating working time in the EU, our operational definition was selected to ensure comparability with prior epidemiological studies and to capture a broader range of biologically relevant night-time exposure. Incorporating such definitions into research studies helps align epidemiological research with occupational health policy and supports evidence-based recommendations for safer scheduling practices.

In conclusion, the EPHOR-NIGHT cohort was created with the aim of providing a holistic understanding of the complex interactions between night shift work exposures, biological pathways and health outcomes. Through the vast number of data elements collected for participants in this cohort, the research that leverages these data will contribute new knowledge on mechanisms and health outcomes within night shift workers, which will be crucial to identifying actionable strategies to mitigate health risks in this vulnerable population.

## Collaboration

Access to study data is possible through a completed data request form, available at https://www.isglobal.org/en/-/ephor. The request form is also included in online supplemental appendix 8. Participants are required to adhere to the specified collaboration requirements, namely include two participants per centre where data are used from, no overlapping topics, relevant human subjects review and review by the steering committee.

## Supplementary material

10.1136/bmjopen-2025-106090 online supplemental appendix 1

10.1136/bmjopen-2025-106090 online supplemental appendix 2

10.1136/bmjopen-2025-106090 online supplemental appendix 3

10.1136/bmjopen-2025-106090 online supplemental appendix 4

10.1136/bmjopen-2025-106090 online supplemental appendix 5

10.1136/bmjopen-2025-106090 online supplemental appendix 6

10.1136/bmjopen-2025-106090 online supplemental appendix 7

10.1136/bmjopen-2025-106090 online supplemental appendix 8

## Data Availability

Data are available upon reasonable request.
