# Supplementary figures and images for: Exposome project for health and occupational research night shift cohort (EPHOR-NIGHT): a unique resource to advance research on night shift work and chronic disease
Source: BMJ Open. 2025 Dec 5;15(12):e106090. doi: 10.1136/bmjopen-2025-106090 (PMC12684079; doi:10.1136/bmjopen-2025-106090)

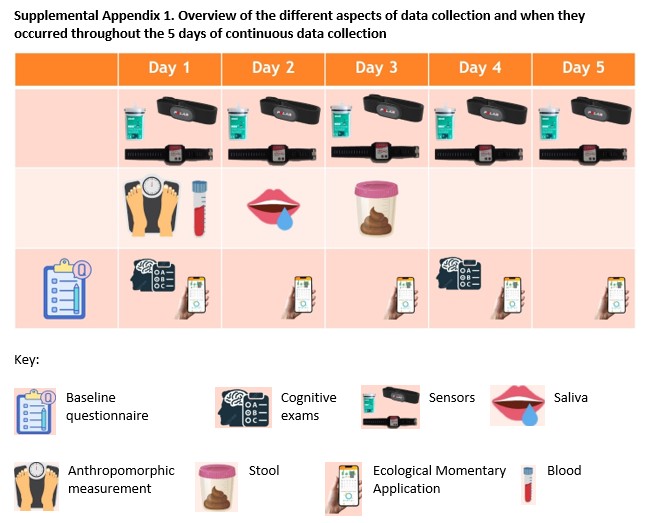

Supplement: online supplemental appendix 1 [file bmjopen-15-12-s001.jpg]
