# Supplementary material for: Exposome project for health and occupational research night shift cohort (EPHOR-NIGHT): a unique resource to advance research on night shift work and chronic disease
Source: BMJ Open. 2025 Dec 5;15(12):e106090. doi: 10.1136/bmjopen-2025-106090 (PMC12684079; doi:10.1136/bmjopen-2025-106090)
Supplement: online supplemental appendix 2 [file bmjopen-15-12-s002.pdf]

# WP7 BASELINE

Please complete the survey below.

Thank you!

---

Please indicate your date of birth (DD-MM-YYYY). __________________________________

WE WILL NOW ASK YOU GENERAL QUESTIONS ABOUT DEMOGRAPHICS AND RESIDENCE.

Please indicate your sex.
- ◯ Male
- ◯ Female

Please indicate your height (in centimeters). Note: if you do not know, please approximate. __________________________________

Please indicate your weight (in kilograms). Note: if you do not know, please approximate. __________________________________

Were you born in Spain? (updated to XX country based on location)
- ◯ Yes
- ◯ No

What country were you born in? __________________________________

Were your parents born in XX country?
- ◯ Yes
- ◯ No

Think of one of your parents first, which country were they born in? __________________________________

Think of your other parent, which country were they born in? __________________________________

Please indicate your address: street name and house or apartment number __________________________________

Which city or town do you live in? __________________________________

Please indicate your postal code. __________________________________

For how many years have you lived at your current address? (please round to the nearest whole number. If you have lived there less than 6 months, please indicate 0) __________________________________

Please indicate your marital status.
- ◯ Married or living together with partner
- ◯ Divorced/separated
- ◯ Widow/widower
- ◯ In a relationship but not living together
- ◯ Single
- ◯ Other

| | |
|---|---|
| Please indicate the highest level of education you have achieved. | ◯ < Secondary education completed<br>◯ Secondary education completed<br>◯ University degree completed<br>◯ Graduate degree completed |
| Please indicate the highest level of education that your partner has achieved? | ◯ < Secondary education completed<br>◯ Secondary education completed<br>◯ University degree completed<br>◯ Graduate degree completed |
| WE WILL NOW ASK YOU QUESTIONS ABOUT YOUR CURRENT WORK AND YOUR WORK HISTORY. | |
| Do you always work the same shift or do you rotate? | ◯ Permanent (always the same) day<br>◯ Permanent (always the same) night<br>◯ Rotate |
| Have you ever worked night shifts in the past? | ◯ Yes<br>◯ No |
| What direction of rotation do you work? | ◯ Forward (morning/afternoon/ evening/night)<br>◯ Backward (afternoon/evening/ morning/night)<br>◯ Other |
| What is the rate of rotation? | ◯ Daily change<br>◯ Change every 2-4 days<br>◯ Change every week<br>◯ Change every 2-3 weeks |
| When does your day shift start? | ______________________ |
| When does your day shift end? | ______________________ |
| When does your night shift start? | ______________________ |
| When does your night shift end? | ______________________ |
| What is the main reason for working night shifts? | ◯ Is part of my job<br>◯ Pleasant schedule, fits my rhythm<br>◯ Calmer work<br>◯ Financial compensation<br>◯ Other |
| How often do you work a long shift (defined as 12 or more hours at a time)? | ◯ Never<br>◯ Once a month or fewer<br>◯ Every other week<br>◯ Once a week<br>◯ Multiple times a week<br>◯ Always |

| | |
|---|---|
| How many consecutive night shifts do you work on average? | ◯ Only work one night and then have a day off<br>◯ 2 consecutive nights<br>◯ 3 consecutive nights<br>◯ 4 consecutive nights<br>◯ 5 or more consecutive nights |
| On average, how many night shifts do you work per week? | ◯ Only work one night per week<br>◯ 2 nights per week<br>◯ 3 nights per week<br>◯ 4 nights per week<br>◯ 5 or more nights per week |
| Do you have the ability to self-roster? | ◯ Yes<br>◯ No |
| How many days of rest (days off from work) do you have per week? | ◯ 0<br>◯ 1<br>◯ 2<br>◯ 3<br>◯ 4 or more |
| At what age did you start working night shifts? | __________________________________ |
| For how many years have you worked a night shift schedule for some or all of the year? (Add up all the periods you have worked night shift and indicate this number- if it is less than 1 year, count it as 1 whole year) | __________________________________ |
| How much travel time do you have on average between your home and work (one way travel time)? | ◯ Less than 15 minutes<br>◯ 15-29 minutes<br>◯ 30-44minutes<br>◯ 45-59 minutes<br>◯ 1-1.5 hours<br>◯ More than 1.5 hours |
| How many hours per week do you work under your contract? | ◯ Less than 35<br>◯ 35-40<br>◯ 41-48<br>◯ 49-54<br>◯ 55 or more |
| WE WILL NOW ASK YOU QUESTIONS ABOUT YOUR SLEEP PREFERENCES AND HABITS. | |
| One hears about "morning" and "evening" types of people. Which ONE of these types do you consider yourself to be? (Morning types are early risers, perform mentally and physically at their best in the morning hours, and go to bed early in the evening. Evening types stay up late at night, rise at a later time in the morning, and perform best mentally and physically in the late afternoon or evening.) | ◯ Definitely a "morning" type<br>◯ Rather more a "morning" type than an "evening" type<br>◯ Rather more an "evening" type than a "morning" type<br>◯ Definitely an "evening" type |
| During the past month, when have you usually gone to bed on a workday? | __________________________________ |

| | |
|---|---|
| During the past month, how long (in minutes) has it ususally taken you to fall asleep? | ◯ 15 minutes or fewer<br>◯ 16-30 minutes<br>◯ 31-60 minutes<br>◯ 60 minutes or more |
| During the past month, when have you usually gotten up in on a workday? | ______________________________ |
| During the past month, how many hours of actual sleep did you get each day (this may be different than the number of hours you spend in bed). | ◯ More than 7 hours<br>◯ 6-7 hours<br>◯ 5-6 hours<br>◯ Fewer than 5 hours |

**During the past month, how often have you had trouble sleeping because you:**

| | Not during past month | Less than once a week | Once or twice a week | Three or more times a week |
|---|---|---|---|---|
| Cannot get to sleep within 30 minutes | ◯ | ◯ | ◯ | ◯ |
| Wake up in the middle of the night or early morning | ◯ | ◯ | ◯ | ◯ |
| Have to get up to use the bathroom | ◯ | ◯ | ◯ | ◯ |
| Cannot breathe comfortably | ◯ | ◯ | ◯ | ◯ |
| Cough or snore loudly | ◯ | ◯ | ◯ | ◯ |
| Feel too cold | ◯ | ◯ | ◯ | ◯ |
| Feel too hot | ◯ | ◯ | ◯ | ◯ |
| Had bad dreams | ◯ | ◯ | ◯ | ◯ |
| Have pain | ◯ | ◯ | ◯ | ◯ |
| Other reasons | ◯ | ◯ | ◯ | ◯ |

| | |
|---|---|
| During the past month, how would you rate your sleep quality overall? | ◯ Very good<br>◯ Fairly good<br>◯ Fairly bad<br>◯ Very bad |
| During the past month, how often have you taken medicine (prescribed or "over the counter") to help you sleep? | ◯ Not during the past month<br>◯ Less than once a week<br>◯ Once or twice a week<br>◯ Three or more times a week |
| During the past month, how often have you had trouble staying awake while driving, eating meals, or engaging in social activity? | ◯ Not during the past month<br>◯ Less than once a week<br>◯ Once or twice a week<br>◯ Three or more times a week |
| During the past month, how much of a problem has it been for you to keep up enough enthusiasm to get things done? | ◯ No problem at all<br>◯ Only a very slight problem<br>◯ Somewhat of a problem<br>◯ A very big problem |

| | |
|---|---|
| During the week how often do you take naps? | ◯ Never<br>◯ 1-2 times<br>◯ 3-7 days a week |
| How long does the nap take on average per day? | ◯ Less than 30 minutes<br>◯ 30 minutes-1 hour<br>◯ More than 1 hour |
| Are you able to take naps DURING a night shift? | ◯ Yes<br>◯ No |
| How dark is your bedroom when you sleep at night? | ◯ Not dark at all (daylight)<br>◯ A little dark (dim)<br>◯ Dark<br>◯ Very dark (cannot see hand extended in front of face) |
| How dark is your bedroom when you sleep between two night shifts? | ◯ Not dark at all (daylight)<br>◯ A little dark (dim)<br>◯ Dark (can see shadows)<br>◯ Very dark (cannot see hand extended in front of face) |
| WE WILL NOW ASK QUESTIONS ABOUT YOUR LIFESTYLE (SMOKING, ALCOHOL, DIET AND EXERCISE) | |
| Have you ever smoked regularly, that is, at least one cigarette per day, a couple of cigars a week, or ecigarette use each day for six months or more? | ◯ Yes<br>◯ No |
| Do you currently smoke or use smokeless tobacco? | ◯ Yes<br>◯ No |
| How many cigarettes do you currently smoke on average per week? (if none, indicate 0) | ____________________ |
| How many ecigarettes do you currently smoke on average per week? (if none, indicate 0) | ____________________ |
| How many cigars do you currently smoke on average per week? (if none, indicate 0) | ____________________ |
| How many grams of pipe tobacco (with a pipe having on average 2 grams of tobacco) do you currently smoke on average per week? (if none, indicate 0) | ____________________ |
| How many grams of smokeless tobacco (typically one uses approximately 1 gram at a time) do you currently use on average per week? (if none, indicate 0) | ____________________ |
| Do you currently drink alcohol? | ◯ No, I quit<br>◯ No, I do not currently drink and never drank<br>◯ Yes |
| How many years ago did you quit drinking alcohol? | ____________________ |

The next set of questions will ask about alcohol use during the past year.

| | |
|---|---|
| How often do you have a drink containing alcohol? | ◯ Never<br>◯ Monthly or less<br>◯ 2-4 times a month<br>◯ 2-4 times a week<br>◯ 4 or more times a week |

A "standard" drink is any drink that contains about 0.6 fluid ounces or 14 grams of pure alcohol. This figure shows different drink sizes, each containing approximately the same amount of alcohol and counting as a single standard drink. The examples serve as starting point for comparison.

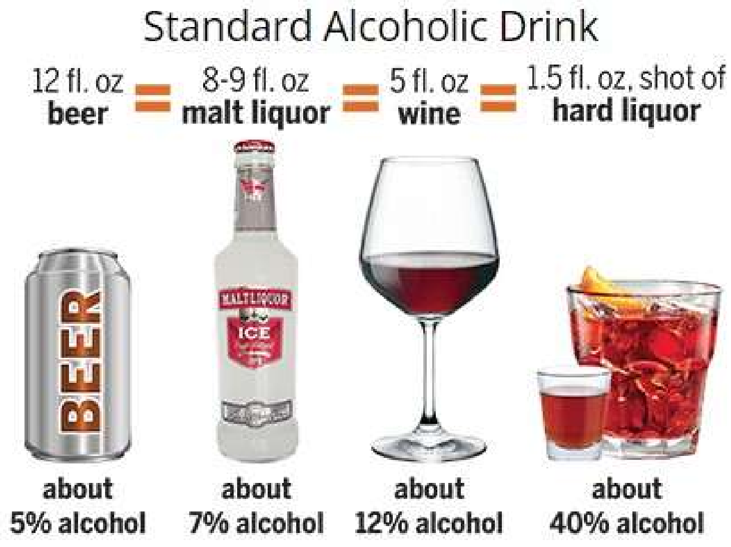


| | |
|---|---|
| How many standard drinks containing alcohol do you have on a typical day when drinking? (one standard drink is equivalent to 12 fluid oz of beer or 5 fluid oz of wine or 1.5 fluid oz of hard liquor) | ◯ 1 or 2<br>◯ 3 to 4<br>◯ 5 to 6<br>◯ 7 to 9<br>◯ 10 or more |
| How often did you have 6 or more drinks on one occasion? | ◯ Never<br>◯ Less than monthly<br>◯ Monthly<br>◯ Weekly<br>◯ Daily or almost daily |

THE FOLLOWING QUESTIONS ARE ABOUT THE TIME YOU SPENT BEING PHYSICALLY ACTIVE IN THE LAST 7 DAYS. THIS INCLUDE ACTIVITIES YOU DO AT WORK, AS PART OF YOUR HOUSE AND YARD WORK, TO GET FROM PLACE TO PLACE, AND IN YOUR SPARE TIME FOR RECREATION, EXERCISE OR SPORT.

| | |
|---|---|
| During the last 7 days, on how many days did you do vigorous physical activitieis like heavy lifting, digging, aerobics, or fast bicycling? (Think about only those physical activities that you did for at least 10 minutes at a time.) | ◯ 1 day per week<br>◯ 2 days per week<br>◯ 3 days per week<br>◯ 4 days per week<br>◯ 5 days per week<br>◯ 6 days per week<br>◯ 7 days per week<br>◯ none |

How much time in total did you usually spend on ONE of those days doing vigorous physical activities?

How many minutes? ______________________________

Again, think only about those physical activities that you did for at least 10 minutes at a time. During the last 7 days, on how many days did you do moderate physical activities like carrying light loads, bicycling at a regular pace, or doubles tennis? Do not include walking.

- ◯ 1 day per week
- ◯ 2 days per week
- ◯ 3 days per week
- ◯ 4 days per week
- ◯ 5 days per week
- ◯ 6 days per week
- ◯ 7 days per week
- ◯ none

How much time in total did you usually spend on ONE of those days doing moderate physical activities?

How many minutes?

__________________________________

During the last 7 days, on how many days did you walk for at least 10 minutes at a time?

- ◯ 1 day per week
- ◯ 2 days per week
- ◯ 3 days per week
- ◯ 4 days per week
- ◯ 5 days per week
- ◯ 6 days per week
- ◯ 7 days per week
- ◯ none

How much time did you usually spend walking on one of those days?

How many minutes?

__________________________________

The last question is about the time you spent sitting on a workday, at home, while doing course work and during leisure time. This includes time spent sitting at a desk, visiting friends, reading, traveling on a bus or sitting or lying down to watch television.

How many minutes?

__________________________________

THE FOLLOWING QUESTIONS ASK ABOUT YOUR DIET.

## With what frequency do you eat the following types of foods?

| | One or more times a day | 4-6 times a week | 3 x per week | Once or twice a week | Less than once a week | Never | Do not know |
|---|---|---|---|---|---|---|---|
| Fresh fruit (excluding juices) | ◯ | ◯ | ◯ | ◯ | ◯ | ◯ | ◯ |
| Red meat (beef, pork, lamb) | ◯ | ◯ | ◯ | ◯ | ◯ | ◯ | ◯ |
| Poultry | ◯ | ◯ | ◯ | ◯ | ◯ | ◯ | ◯ |
| Eggs | ◯ | ◯ | ◯ | ◯ | ◯ | ◯ | ◯ |
| Fish | ◯ | ◯ | ◯ | ◯ | ◯ | ◯ | ◯ |
| Pasta, rice, bread, cereals | ◯ | ◯ | ◯ | ◯ | ◯ | ◯ | ◯ |
| Vegetables | ◯ | ◯ | ◯ | ◯ | ◯ | ◯ | ◯ |
| Beans and legumes | ◯ | ◯ | ◯ | ◯ | ◯ | ◯ | ◯ |

| | | | | | | | |
|---|---|---|---|---|---|---|---|
| Nuts | ◯ | ◯ | ◯ | ◯ | ◯ | ◯ | ◯ |
| Processed meats including sausages and cold cuts | ◯ | ◯ | ◯ | ◯ | ◯ | ◯ | ◯ |
| Dairy products (milk, cheese, yogurt) | ◯ | ◯ | ◯ | ◯ | ◯ | ◯ | ◯ |
| Sweets (cookies, pastries, jams, cereals with sugar, candies) | ◯ | ◯ | ◯ | ◯ | ◯ | ◯ | ◯ |
| Sodas with sugar | ◯ | ◯ | ◯ | ◯ | ◯ | ◯ | ◯ |
| Fast food (fried chicken, sandwiches, pizzas, burgers) | ◯ | ◯ | ◯ | ◯ | ◯ | ◯ | ◯ |
| Snacks or savory finger foods (chips, crackers | ◯ | ◯ | ◯ | ◯ | ◯ | ◯ | ◯ |
| Drink 100% fruit or vegetable juice | ◯ | ◯ | ◯ | ◯ | ◯ | ◯ | ◯ |

How many times per day do you eat fresh fruit?

◯ 1
◯ 2
◯ 3
◯ 4
◯ 5
◯ 6
◯ 7
◯ 8
◯ 9
◯ 10

How many times per day do you eat green leafy salad or other types of vegetables?

◯ 1
◯ 2
◯ 3
◯ 4
◯ 5
◯ 6
◯ 7
◯ 8
◯ 9
◯ 10

How many times per day do you drink 100% fruit or vegetable juice?

◯ 1
◯ 2
◯ 3
◯ 4
◯ 5
◯ 6
◯ 7
◯ 8
◯ 9
◯ 10

What type of dairy do you generally consume?

◯ Skim/fat free
◯ Low fat
◯ Full fat
◯ Do not know

| | |
|---|---|
| What portion of your grains (rice, bread, crackers, pasta, oatmeal, etc.) are whole grains (brown rice, brown bread, whole wheat pasta, etc)? | ◯ Less than half<br>◯ Half<br>◯ More than half<br>◯ All of my grains are whole grains<br>◯ Do not know |
| What time to you generally eat breakfast? | __________________________ |
| What time do you generally eat lunch? | __________________________ |
| What time do you generally eat dinner? | __________________________ |
| Which meal is your largest meal of the day? | ◯ Breakfast<br>◯ Lunch<br>◯ Dinner<br>◯ Do not know |
| Have you ever received guidence about diet or meal timing at your workplace? | ◯ Yes<br>◯ No<br>◯ Do not know |
| WE WILL NOW ASK QUESITONS ABOUT YOUR MEDICAL HISTORY AND MEDICATION USE. | |
| Have you ever had a pregnancy that lasted 24 weeks or more? | ◯ Yes<br>◯ No |
| Have you had a period in the last 12 months? | ◯ Yes<br>◯ No |
| If no, what statement best describes the reason you have not had a period in the last 12 months? | ◯ Menopause<br>◯ Hysterectomy<br>◯ Ovaries removed<br>◯ Currently pregnant<br>◯ Currently breast feeding<br>◯ Taking birth control e.g. hormonal IUD, hormonal contraceptives, contraceptive implants<br>◯ Chemotherapy<br>◯ Other |

## Has your doctor ever told you you had any of the following conditions or diseases?

| | Yes | No | Do not know |
|---|---|---|---|
| Myocardial infarction | ◯ | ◯ | ◯ |
| Cardiac arrhythmia | ◯ | ◯ | ◯ |
| Stroke | ◯ | ◯ | ◯ |
| High cholesterol/triglycerides | ◯ | ◯ | ◯ |
| Hypertension | ◯ | ◯ | ◯ |

| | Yes | No | Do not know |
|---|---|---|---|
| COPD (chronic bronchitis, emphysema) | ◯ | ◯ | ◯ |
| Asthma | ◯ | ◯ | ◯ |
| Diabetes | ◯ | ◯ | ◯ |
| Cancer | ◯ | ◯ | ◯ |
| Depression | ◯ | ◯ | ◯ |
| Anxiety | ◯ | ◯ | ◯ |
| Neurological condition (head trauma, epilepsy, dementia, etc.) | ◯ | ◯ | ◯ |

What type of cancer(s) have you been diagnosed with?

__________________________________________

During the past year have you regularly taken any medications including prescription or over the counter?

◯ Yes
◯ No
◯ Do not know

**During the past 24 hours, have you taken any of the medications in the list below?**

| | Yes | No | Do not know |
|---|---|---|---|
| Aspirin or some other non-steroidal anti-inflammatory NSAID (such as ibuprofen, diclofenac, piroxicam, etc), to treat any condition or to treat pain? | ◯ | ◯ | ◯ |
| Any medications to treat heart disease or hypertension (such as type b-blockers, diuretics, MAO inhibitors, calcium blockers)? | ◯ | ◯ | ◯ |
| Any medication to treat depression (such as Prozac, Zoloft, Paxil, Colexa, Elavil, Tofranil, Valium, Xanax, Librium, etc.)? | ◯ | ◯ | ◯ |
| Melatonin supplements? | ◯ | ◯ | ◯ |
| Any other medicines to treat insomnia besides melatonin (such as hypnotics)? | ◯ | ◯ | ◯ |
| Lipid lowering medication (such as statins)? | ◯ | ◯ | ◯ |
| Diabetes medications (such as metformin, sulfonylurea, insulin)? | ◯ | ◯ | ◯ |

## Over the last 2 weeks, how often have you been bothered by the following problems?

| | Not at all | Several days | Over half the days | Nearly every day |
|---|---|---|---|---|
| 1. Feeling nervous, anxious, or on edge | ◯ | ◯ | ◯ | ◯ |
| 2. Not being able to stop or control worrying | ◯ | ◯ | ◯ | ◯ |
| 3. Worrying too much about different things | ◯ | ◯ | ◯ | ◯ |
| 4. Trouble relaxing | ◯ | ◯ | ◯ | ◯ |
| 5. Being so restless that it's hard to sit still | ◯ | ◯ | ◯ | ◯ |
| 6. Becoming easily annoyed or irritable | ◯ | ◯ | ◯ | ◯ |
| 7. Feeling afraid as if something awful might happen | ◯ | ◯ | ◯ | ◯ |
| If you checked off any problems, how difficult have these made it for you to do your work, take care of things at home, or get along with other people? | ◯ | ◯ | ◯ | ◯ |

## Over the last 2 weeks how often have you been bothered by any of the following problems?

| | Not at all | Several days | More than half the days | Nearly every day |
|---|---|---|---|---|
| 1. Little interest or pleasure in doing things | ◯ | ◯ | ◯ | ◯ |
| 2. Feeling down, depressed, or hopeless | ◯ | ◯ | ◯ | ◯ |
| 3. Trouble falling or staying asleep, or sleeping too much | ◯ | ◯ | ◯ | ◯ |
| 4. Feeling tired or having little energy | ◯ | ◯ | ◯ | ◯ |
| 5. Poor appetite or overeating | ◯ | ◯ | ◯ | ◯ |
| 6. Feeling bad about yourself or that you are a failure or have let yourself or your family down | ◯ | ◯ | ◯ | ◯ |
| 7. Trouble concentrating on things, such as reading the newspaper or watching television | ◯ | ◯ | ◯ | ◯ |
| 8. Moving or speaking so slowly that other people could have noticed? Or the opposite -- being so fidgety or restless that you have been moving around a lot more than usual | ◯ | ◯ | ◯ | ◯ |

| | | | | |
|---|---|---|---|---|
| 9. Thoughts that you would be better off dead or of hurting yourself in some way | ◯ | ◯ | ◯ | ◯ |

**Over the last month, how often have you felt the following things?**

| | Never | Almost Never | Sometimes | Fairly Often | Very Often |
|---|---|---|---|---|---|
| 1. How often have you been upset because of something that happened unexpectedly? | ◯ | ◯ | ◯ | ◯ | ◯ |
| 2. How often have you felt that you were unable to control the important things in your life? | ◯ | ◯ | ◯ | ◯ | ◯ |
| 3. How often have you felt nervous and "stressed"? | ◯ | ◯ | ◯ | ◯ | ◯ |
| 4. How often have you felt confident about your ability to handle your personal problems? | ◯ | ◯ | ◯ | ◯ | ◯ |
| 5. How often have you felt that things were going your way? | ◯ | ◯ | ◯ | ◯ | ◯ |
| 6. How often have you found that you could not cope with all the things that you had to do? | ◯ | ◯ | ◯ | ◯ | ◯ |
| 7. How often have you been able to control irritations in your life? | ◯ | ◯ | ◯ | ◯ | ◯ |
| 8. How often have you felt that you were on top of things? | ◯ | ◯ | ◯ | ◯ | ◯ |
| 9. How often have you been angered because of things that were outside of your control? | ◯ | ◯ | ◯ | ◯ | ◯ |
| 10. How often have you felt difficulties were piling up so high that you coul dnot overocome then? | ◯ | ◯ | ◯ | ◯ | ◯ |

WE WILL NOW ASK YOU QUESTIONS RELATED TO THE COVID-19 PANDEMIC.

Between February 2020 until now did you have one or more of the following symptoms?
Cough
Sore throat
Headache
Muscle ache/pain
Fever
Dyspnoea (difficult breathing)
Reduced or loss of senses of taste and smell
Nausea or vomiting
Diarrhoea
Chest pains
Skin rashes

◯ Yes
◯ No

| | |
|---|---|
| Have you been diagnosed with COVID-l9 on the basis of a test by a doctor/general practitioner /others? | ◯ Yes<br>◯ No |
| Have you ever been hospitalised due to COVID-l9? | ◯ Yes<br>◯ No |
| Do you use any of the following personal protective equipment during your work? | |
| Face covering | ◯ No<br>◯ Yes, it has been face fit tested<br>◯ Yes, it is a home made or surgical mask |
| Gloves | ◯ Yes<br>◯ No |
| Visor | ◯ Yes<br>◯ No |
| In what way has your hours of work changed as a result of COVID-19? | ◯ Increased a lot<br>◯ Increased a little<br>◯ No change<br>◯ Decreased a little<br>◯ Decreased a lot |

**Which of the following control measures are in place where you work?**

| | Yes | No |
|---|---|---|
| Testing of staff/patientes/public | ◯ | ◯ |
| Social distancing | ◯ | ◯ |
| Ventilation | ◯ | ◯ |
| Barriers partial or complete | ◯ | ◯ |
| PPE | ◯ | ◯ |
| Intensified cleaning | ◯ | ◯ |

| | |
|---|---|
| Are you worried about catching the virus? | ◯ Not at all<br>◯ Rarely<br>◯ Sometimes<br>◯ Often<br>◯ Almost always |
| Are you worried that you can't keep your family safe from the virus? | ◯ Not at all<br>◯ Rarely<br>◯ Sometimes<br>◯ Often<br>◯ Almost always |
| How would you rate the changes in your working conditions before and after the beginning of the coronavirus crisis? | ◯ Significant worsening<br>◯ Somewhat worsening<br>◯ Somewhat improving<br>◯ Significant improvement<br>◯ No change |

| | |
|---|---|
| How would you rank the changes in your private life during the COVID-19 crisis? | ◯ Significant worsening<br>◯ Somewhat worsening<br>◯ Somewhat improving<br>◯ Significant improvement<br>◯ No change |
| To what extent have you perceived the coronavirus pandemic as a threat to yourself? | No threat at all to myself — Extreme threat to myself<br>*(Place a mark on the scale above)* |
