# Supplementary material for: Exposome project for health and occupational research night shift cohort (EPHOR-NIGHT): a unique resource to advance research on night shift work and chronic disease
Source: BMJ Open. 2025 Dec 5;15(12):e106090. doi: 10.1136/bmjopen-2025-106090 (PMC12684079; doi:10.1136/bmjopen-2025-106090)
Supplement: online supplemental appendix 3 [file bmjopen-15-12-s003.docx]

**Welcome**

Thank you for participating in NEST-NL and for taking the time to complete this questionnaire. Your answers will help us understand how your personal living environment affects your health.

**Start date of questionnaire (DD/MM/YYYY):**
**Email address:**

**5.1 General**

**How many years have you lived at your current address?**
…… years

**What is your current marital status?**

- Married / registered partnership / cohabiting
- Divorced or separated
- Widowed
- In a relationship but not living together (LAT relationship)
- Single (no partner)
- Living with parent(s)

**What is your current height?**
…….. cm (please round to the nearest whole number, e.g., 165 cm)

**What is your current weight?**
…….. kg
If you are currently pregnant, what was your weight before you became pregnant?
(please round to the nearest whole number, e.g., 76 kg)

**Are you currently pregnant?**

- Yes
- No

**We ask you to measure your hip and waist circumference.**
You can do this with a measuring tape (e.g., a roll-up tape); instructions are given below.

**Hip circumference:**
Measure your hip circumference on bare skin or over light clothing. Stand with your feet together and place the tape measure around the widest part of your lower body (around hips or buttocks). Make sure the tape is horizontal and not too tight. Measure twice and record the average of the two values.
If you are pregnant, you do not need to measure your hip circumference.

**Waist circumference:**
Stand with your feet about 30 cm apart, relaxed and upright, not holding your breath. Measure on bare skin or over light clothing after a normal breath out. Place the tape between the bottom of your lowest rib and the top of your pelvis, horizontally relative to the floor (see Figure 1). If the correct spot is hard to find, measure at navel height. Do not pull the tape too tight. Measure twice and record the average.

**What is your current hip circumference?**
…….. cm (please round to the nearest whole number, e.g., 95 cm)

**What is your current waist circumference?**
…….. cm (please round to the nearest whole number, e.g., 85 cm)

**Smoking**

**Did you smoke at least one cigarette per day around January 1, 2017? And do you currently smoke?**

- Yes, I smoked then and I still (or again) smoke
- Yes, I smoked then but I have since quit
- No, I didn’t smoke then but I do now
- No, I never smoked / I quit before 2017

**How many cigarettes do you currently smoke on average per day?**
[Open field]

**At what age did you stop smoking?**
[Open field]

**Alcohol Consumption**

**On how many days per week did you usually drink alcohol in the past 12 months?**

- I did not drink alcohol in the past 12 months
- Rarely, less than 1 day per week
- 1 day per week
- 2–3 days per week
- 4–5 days per week
- 6–7 days per week

**How many glasses per week of the following types of alcohol did you drink on average in the past 12 months?**

- Beer (1 bottle counts as 1.5 glasses)
- Red wine
- White wine and/or rosé
- Sherry, port, vermouth, etc.
- Spirits / mixed drinks / cocktails / alcopops (e.g. Breezers)

**How often do you drink 6 or more alcoholic drinks on a single occasion?**

- Never
- Less than monthly
- Monthly
- Weekly
- Daily

**Physical Activity**

**Can you indicate in the table below how many hours per week, on average, you spent on the following activities during the past summer and winter?**

| **Activity** | **Summer (hours/week)** | **Winter (hours/week)** |
| --- | --- | --- |
| Cycling (e.g., commuting, leisure, errands) |  |  |
| Walking (e.g., commuting, leisure, golfing, errands) |  |  |
| Housework (e.g., laundry, cleaning, cooking) |  |  |
| Gardening |  |  |
| DIY/handyman tasks |  |  |
| Moderate physical activity (e.g., swimming, yoga, dancing, recreational rowing, doubles tennis) |  |  |
| Vigorous physical activity (that makes you sweat and/or breathe harder, e.g., running, squash, aerobics/step, singles tennis) |  |  |

**Sedentary Behavior (last 7 days)**

**How many minutes per day did you spend sitting during the last seven days in the following situations?**

- On a **workday**: [__] minutes/day
- On a **day off**: [__] minutes/day

**Menstruation**

**Have you had at least one menstrual period since January 1, 2017?**

- Yes
- No

**When was your last menstrual period?**
[Enter month/year]

**What was the reason your menstruation stopped?** (Multiple answers possible)

- Due to use of contraceptives (e.g., hormonal IUD, continuous pill use)
- Pregnancy or breastfeeding
- Spontaneously, menstruation stopped on its own (e.g., menopause)
- After stopping the pill/injection
- Due to surgery on the uterus
- Due to surgery on the ovary(ies)/fallopian tube(s)
- Due to chemotherapy or radiation therapy
- Due to use of medications (e.g., after removal of ovaries or for menopausal symptoms)
- Significant weight loss
- I don’t know
- Other, namely: [Open field]
- Not applicable, I still menstruate

**Pregnancies**

**Were you pregnant on January 1, 2017, or have you had a pregnancy after that date that lasted 24 weeks or longer?**

- Yes
- No

**Contraceptives and Other Hormonal Medications**

**Have you used any of the following contraceptives or other hormonal medications since January 1, 2017?**

- No
- Yes, condoms
- Yes, periodic abstinence
- Yes, the pill (even if used for reasons other than contraception)
- Yes, the injection (e.g., Depo-Provera)
- Yes, contraceptive patches
- Yes, hormonal IUD/Mirena (excluding copper IUD)
- Yes, contraceptive implant
- Yes, contraceptive ring/NuvaRing (excluding diaphragm/cervical cap)
- Yes, a non-hormonal contraceptive (e.g., copper IUD, diaphragm, female condom)
- Yes, a hormone-containing medication not used for contraception or IVF

**Work – General**

**What is your highest completed level of education?**

- Primary school / No education
- Secondary school
- Vocational education (MBO)
- Higher professional education (HBO)
- University bachelor
- University master or higher

**What is your partner’s highest completed level of education?**

- Primary school / No education
- Secondary school
- Vocational education (MBO)
- Higher professional education (HBO)
- University bachelor
- University master or higher

**Which of the following situations applied to you on January 1, 2017?**

- I was employed (salaried work)
- I was self-employed / freelancer
- I was retired (early retirement, AOW, VUT, FPU)
- I was unemployed / looking for work
- I was unable to work (disability)
- I was receiving social assistance
- I was doing volunteer work / informal caregiving
- I was a housewife/househusband
- I was in education / studying

**Have you worked since January 1, 2017?**

- No
- Yes, in salaried employment
- Yes, as a self-employed person / freelancer

**Which job did you have on January 1, 2017?**

- Care assistant
- Nurse
- Other, but still in healthcare: [Open field]
- Other, namely: [Open field]

**Job – Specifics (Job #1)**

**How many hours per week did you work in this job (contract hours)?**
[Open field]

**How many hours per week did you actually work in this job?**
[Open field]

**How many days off (non-working days) did you have per week in this job?**
[Open field]

**How often did you work a long shift (12 hours or more)?**

- 0
- 1
- 2
- 3
- 4 or more

**Frequency of long shifts:**

- Never
- Once per month or less
- Every other week
- Once per week
- Several times per week
- Always

**What best describes the physical demands of this job?**

- Sedentary
- Standing/walking
- Physically demanding

**Did you ever work rotating and/or permanent shifts for 6 months or longer in this job?**

- Yes
- No
- No, only shorter than 6 months

**Which of the following shifts were part of your schedule in this job?**

- Early shift (i.e. starting between 05:00 and 06:59; note: this is not the same as a day shift)
- Evening shift (i.e. at least 1 hour worked after 19:00, ending before midnight)
- Night shift (i.e. at least 1 hour worked between midnight and 05:00)
- Sleep shift (i.e. sleeping at the workplace or only working when woken up if needed)
- None of the above (only day shifts / office hours)

**How were your rotating shifts scheduled?**

- Forward rotating (e.g. day–evening–night)
- Backward rotating (e.g. night–evening–day)
- Always varying
- Other, namely: [Open field]

**How quickly did your shift schedule rotate?**

- Daily
- Every 2–4 days
- Weekly
- Every 2–3 weeks

**Were you able to determine your own shift schedule?**

- No
- Yes

**Early Shifts – Job #1**

*Early shifts are defined as those that* ***start between 05:00 and 06:59 in the morning****. These are not the same as regular day shifts.*

**How many early shifts did you work on average per month in this job?**
[Open field]

- I cannot provide an answer because it varied too much

**Did you work roughly the same number of early shifts in different periods?**

- No, it varied a lot in this job
- Yes, approximately the same across periods
- I don't remember

**How many early shifts did you typically work consecutively?**
[Open field]
(If it varied a lot, e.g. between 0 and 4 per month, enter the average: 2)

**What were the usual start and end times of the early shift?**

- Start time: [hh:mm, e.g. 05:30]
- End time: [hh:mm, e.g. 14:00]

**Early Shifts – Periods within Job #1**

*For each distinct period in which you worked early shifts within this job, please fill in the following:*

**Period 1**

- Start year: [YYYY]
- End year: [YYYY or current date]
- Average number of early shifts per month: [Open field]
- Average number of early shifts in a row: [Open field]
- Start time: [hh:mm]
- End time: [hh:mm]

**Was there a second period in this job where you worked early shifts?**

- No
- Yes

*If yes: repeat as above for Period 2 and subsequent periods if applicable.*

**Evening Shifts – Job #1**

*Evening shifts are defined as those with* ***at least 1 hour of work after 19:00 and ending before midnight****. These are not night shifts.*

**How many evening shifts did you work on average per month in this job?**
[Open field]

- I cannot provide an answer because it varied too much

**Did you work roughly the same number of evening shifts in different periods?**

- No, it varied a lot in this job (e.g. sometimes 3 in a month, sometimes 12, sometimes none)
- Yes, approximately the same across periods
- I don't remember

**How many evening shifts did you typically work consecutively?**
[Open field]
(If it varied a lot, e.g. between 0 and 4 per month, enter the average: 2)

**What were the usual start and end times of the evening shift?**

- Start time: [hh:mm]
- End time: [hh:mm]

**Evening Shifts – Periods within Job #1**

*For each distinct period in which you worked evening shifts within this job, please fill in the following:*

**Period 1**

- Start year: [YYYY]
- End year: [YYYY or current date]
- Average number of evening shifts per month: [Open field]
- Average number of evening shifts in a row: [Open field]
- Start time: [hh:mm]
- End time: [hh:mm]

**Was there another period in this job where you worked evening shifts?**

- No
- Yes

*If yes: repeat as above for Period 2 and subsequent periods if applicable.*

**Night Shifts – Job #1**

*Night shifts are defined as those with* ***at least 1 hour of work between midnight and 05:00 in the morning****. These are not evening shifts.*

**How many night shifts did you work on average per month in this job?**
[Open field]

- I cannot provide an answer because it varied too much

**Did you work roughly the same number of night shifts in different periods?**

- No, it varied a lot in this job (e.g., sometimes 3 in a month, sometimes 12, sometimes none)
- Yes, approximately the same across periods
- I don't remember

**How many night shifts did you typically work consecutively?**
[Open field]
(If it varied a lot, e.g., between 0 and 4 per month, enter the average: 2)

**What were the usual start and end times of the night shift?**

- Start time: [hh:mm]
- End time: [hh:mm]

**Was working night shifts voluntary?**

- No
- Yes

**Night Shifts – Periods within Job #1**

*For each distinct period in which you worked night shifts within this job, please fill in the following:*

**Period 1**

- Start year: [YYYY]
- End year: [YYYY or current date]
- Average number of night shifts per month: [Open field]
- Average number of night shifts in a row: [Open field]
- Start time: [hh:mm]
- End time: [hh:mm]
- Was working night shifts voluntary?
  - No
  - Yes

**Was there another period in this job where you worked night shifts?**

- No
- Yes

*If yes: repeat as above for Period 2 and subsequent periods if applicable.*

**Sleep Shifts – Job #1**

*Sleep shifts are defined as shifts where you* ***sleep at your workplace****, or only work if needed and are woken up.*

**How many sleep shifts did you work on average per month in this job?**
[Open field]

- I cannot provide an answer because it varied too much

**Did you work roughly the same number of sleep shifts in different periods?**

- No, this varied a lot in this job (e.g., sometimes 3 in a month, sometimes 12, sometimes none)
- Yes, approximately the same across periods
- I don’t remember

**How many sleep shifts did you typically work consecutively?**
[Open field]
(If it varied a lot, e.g., between 0 and 4 per month, enter the average: 2)

**What were the usual start and end times of the sleep shift?**

- Start time: [hh:mm]
- End time: [hh:mm]

**During your sleep shifts, how much of the time were you awake/working?**

- I was rarely woken up and usually slept well
- I was rarely woken up but usually lay awake
- I was sometimes woken up and usually slept well in between
- I was sometimes woken up but usually lay awake
- I was awake / working most of the night

**Sleep Shifts – Periods within Job #1**

*For each distinct period in which you worked sleep shifts within this job, please fill in the following:*

**Period 1**

- Start year: [YYYY]
- End year: [YYYY or current date]
- Average number of sleep shifts per month: [Open field]
- Average number of sleep shifts in a row: [Open field]
- Start time: [hh:mm]
- End time: [hh:mm]
- Portion of the night awake/working (select one as above)

**Was there another period in this job where you worked sleep shifts?**

- No
- Yes

*If yes: repeat as above for Period 2 and subsequent periods if applicable.*

**End of Job #1**

**Is this your current job?**

- Yes
- No, I stopped working in this job in [year]

**Did you have another paid job after this one (after January 1, 2017)?**

- Yes
- No

**What was that job (after January 1, 2017)?**

*If you had more than one job during that time, describe the job to which you devoted the most time.*

- Care assistant
- Nurse
- Other, but still in healthcare: [Open field]
- Other, namely: [Open field]

This leads into the **Job-Specific Details (Job #2 and subsequent jobs)**, which follows the same format as for Job #1 (contract hours, shift types, timing, etc.).

**Sleep Habits and Chronotype**

**How many hours of sleep do you usually get on a workday?**
[Open field] hours

**How many hours of sleep do you usually get on a day off (non-working day)?**
[Open field] hours

**Do you have trouble falling asleep at night?**

- Never
- Rarely
- Sometimes
- Often
- Always

**Do you wake up during the night and have trouble going back to sleep?**

- Never
- Rarely
- Sometimes
- Often
- Always

**Do you feel tired or drowsy during the day?**

- Never
- Rarely
- Sometimes
- Often
- Always

**In general, how satisfied are you with your sleep?**

- Very dissatisfied
- Dissatisfied
- Neutral
- Satisfied
- Very satisfied

**Chronotype (Morningness–Eveningness):**

**What best describes you?**

- Definitely a morning person
- More a morning than an evening person
- More an evening than a morning person
- Definitely an evening person
- Neither

**If you could plan your day freely, what time would you go to bed?**
[hh:mm]

**If you could plan your day freely, what time would you wake up?**
[hh:mm]

**Health Status and Medication Use**

**How would you rate your current general health?**

- Excellent
- Very good
- Good
- Fair
- Poor

**Do you currently suffer from any of the following conditions (diagnosed by a doctor)?**
*(Check all that apply)*

- High blood pressure
- Heart disease
- Stroke or TIA
- Diabetes (type 1 or 2)
- High cholesterol
- Asthma
- Chronic bronchitis or COPD
- Cancer
- Thyroid disorder
- Depression
- Anxiety disorder
- Burnout or stress-related complaints
- Chronic fatigue
- Rheumatism or arthritis
- Osteoporosis
- Other, namely: [Open field]
- None of the above

**Are you currently taking any medications on a regular basis (at least once a week)?**

- Yes
- No

**If yes, please indicate for which conditions:**
*(Check all that apply)*

- High blood pressure
- Heart disease
- Diabetes
- High cholesterol
- Depression
- Anxiety
- Sleep problems
- Pain
- Contraception
- Hormone therapy
- Other, namely: [Open field]

**Menopausal Symptoms and Hormone Use**

**Have you ever experienced menopausal symptoms (e.g., hot flashes, night sweats, mood changes, sleep disturbances)?**

- No
- Yes, mild symptoms
- Yes, moderate symptoms
- Yes, severe symptoms

**Are you currently experiencing menopausal symptoms?**

- No
- Yes, mild symptoms
- Yes, moderate symptoms
- Yes, severe symptoms

**Have you ever used hormone replacement therapy (HRT)?**

- No
- Yes, I am currently using it
- Yes, I used it in the past

**If you have used or are using HRT, for how many years in total?**
[Open field]

**For what reason(s) have you used HRT?**
*(Check all that apply)*

- Menopausal symptoms
- Prevention of osteoporosis
- Other, namely: [Open field]

**Contact and Comments**

**Would you be willing to participate in follow-up research related to this questionnaire?**

- Yes
- No

**Do you have any comments or feedback about this questionnaire or your participation in the study?**
[Open field]
