# Supplementary material for: Exposome project for health and occupational research night shift cohort (EPHOR-NIGHT): a unique resource to advance research on night shift work and chronic disease
Source: BMJ Open. 2025 Dec 5;15(12):e106090. doi: 10.1136/bmjopen-2025-106090 (PMC12684079; doi:10.1136/bmjopen-2025-106090)
Supplement: online supplemental appendix 4 [file bmjopen-15-12-s004.pdf]

National Research Centre for the Working Environment

Background questionaire, English

Type of answer to the question	Component	Response options if applicable	Documentation
**General**			
Thank you for participating in the research project "1001 nigths". The background questionnaire is completed once before the start of the experiment.			
Choice of date in calendar (dd-mm-yyyy)	1. When are you born? (DD-MM-YYYY).		
Checkbox menu	2. What is your marital status?	Single; In a relationship; Married; Divorced/separated; Widow/widower; Other/do not wish to answer	
Checkbox menu	3. Are you living with a spouse or partner?	Yes; No	
Checkbox menu	3.1 At what time of the day does your spouse / partner primarily work?	Day work; Evening work; Night work; Not applicable	
Drop-down list	4. How many children are there in your household? (including stepchildren and children in shared custody)	0; 1; 2; 3; 4; 5; 6+	
Drop-down list	4.1 How many of the children are younger than 7 years old?	0; 1; 2; 3; 4; 5; 6+	
Checkbox menu	5. What is the highest level of education you have achieved.	Danish primary and lower secondary school or equivalent; Danish upper secondary school (STX, HHX, HTX, HF); Danish vocational education; Short higher education (< 3 years); Medium length higher education (3-4 years); Long higher education (> 4 years)	
Checkbox menu	5.1 What is the highest level of education that	Danish primary and lower secondary school or equivalent; Danish upper	

	your partner has achieved.	secondary school (STX, HHX, HTX, HF); Danish vocational education; Short higher education (< 3 years); Medium length higher education (3-4 years); Long higher education (> 4 years)	
Checkbox menu	6. How would you best describe your origin?	Asian; African; South American; Middle Eastern; European; Other; I prefer not to answer	
Text field	6.1 How would you best describe your origin?		
**Working life**			
Text field	7. How many years have you been working at your current workplace?		
	Job and motivation<br>*Some of the following questions will be about your working hours. Indicate time of day in the following way:*<br>*11.30 p.m. is referred to as:*<br>*HOUR: 23*<br>*MINUTE: 30*<br>*7.00 a.m. is referred to as:*<br>*HOUR: 07*<br>*MINUTE: 00*		
Checkbox menu	8. At what time of day do you usually work in your main occupation?	Permanent daytime work (mainly between 6:00 and 18:00); Permanent evening work (mainly between 15:00 and 24:00); Permanent night work (mainly between midnight and 5:00); Changing working hours with night work; Changing working hours without night work	
Text fields (hour and minute)	9. When does a typical day shift start?		
Text fields (hour and minute)	9. When does a typical day shift end?		
Text fields (hour and minute)	10. When does a typical evening shift start?		
Text fields (hour and minute)	10. When does a typical evening shift end?		

Text fields (hour and minute)	11. When does a typical night shift start?		
Text fields (hour and minute)	11. When does a typical night shift end?		
Checkbox menu	12. How often do you work a long shift (defined as 12 or more hours)?	Never; Once a month or fewer; Every other week; Once a week; Multiple times a week; Always	
Checkbox menu	13. Have you ever worked night shifts with at least 3 night shifts per month? It is considered a night shift, when you a least have 3 hours between midnight and 6 AM.	Yes; No; Don't know	
Drop-down list	13.1 When did you start working night shifts?	1940, 1941, 1942…2021, 2022, 2023	
Text field	13.2 For how many years of your life have you had night shifts?		
Text field	13.3 How many consecutive night shifts do you prefer to have?		
Checkbox menu	13.4 How many consecutive night shifts do you work on average?	Only one night shift in a row; 2 consecutive night shifts; 3 consecutive night shifts; 4 consecutive night shifts; 5 or more consecutive night shifts	
Checkbox menu	13.5 On average, how many night shifts do you work per week?	Only work one night shifts per week; 2 night shifts per week; 3 night shifts per week; 4 night shifts per week; 5 or more night shifts per week	
Checkbox menu	13.6 Are you able to take naps during a night shift?	Yes; No	
Nummeric scale	13.7 All things considered, how strenuous do you find it having night shifts? Choose 1 for not at all strenuous and 7 for very strenuous.	Whole numbers from1-7	

Checkbox menu	14. Do you have influence on your own working hours?	Yes; No	
Drop-down list	15. What is your mean number of days off per week?	0; 1; 2; 3; 4 or more	
Drop-down list	16. How much travel time do you have on average between your home and work (one way commu-ting time)?	Less than 15 minutes; 15-29 minutes; 30-44 minutes; 45-59 minutes; 1-1,5 hours; More than 1,5 hours	
Text field	17. How many hours per week do you work in your main occupation (including additional hours, if any)?		
Checkbox menu	18. Do you experience any conflicts between your work and private life, so that you would rather be in both places at the same time?	Yes, often; Yes, regularly; Rarely; No, never	Copenhagen Psychosocial Questionnaire (COPSOQ-II)
**Sleep**			
Checkbox menu	19. The following questions are about your sleep the past four weeks. Do not count in holidays. Tick one box in each line. How often…	Never; Seldom; Sometimes (once or twice a month); Fairly often (once or more per week); Always (almost every day)	Karolinska Sleep Questionnaire
	Have you had difficulties falling asleep?	Never; Seldom; Sometimes (once or twice a month); Fairly often (once or more per week); Always (almost every day)	Karolinska Sleep Questionnaire
	Have you had difficulties awakening?	Never; Seldom; Sometimes (once or twice a month); Fairly often (once or more per week); Always (almost every day)	Karolinska Sleep Questionnaire
	Have you wakened too early, unable too fall asleep again?	Never; Seldom; Sometimes (once or twice a month); Fairly often (once or more per week); Always (almost every day)	Karolinska Sleep Questionnaire
	Have you felt that you were not rested when you woke up?	Never; Seldom; Sometimes (once or twice a month); Fairly often	Karolinska Sleep Questionnaire

		(once or more per week); Always (almost every day)	
	Have you experienced repeated awakenings with difficulties going back to sleep?	Never; Seldom; Sometimes (once or twice a month); Fairly often (once or more per week); Always (almost every day)	Karolinska Sleep Questionnaire
	Have you experienced disturbed/restless sleep?	Never; Seldom; Sometimes (once or twice a month); Fairly often (once or more per week); Always (almost every day)	Karolinska Sleep Questionnaire
	Have you felt exhaustion at the awakening?	Never; Seldom; Sometimes (once or twice a month); Fairly often (once or more per week); Always (almost every day)	Karolinska Sleep Questionnaire
Checkbox menu	20. How is your sleep in general?	Excellent; Very good; Good; Less good; Bad	Karolinska Sleep Questionnaire
Checkbox menu	21. During the past four weeks, how many hours of sleep did you get each day?	5 hours or fewer; 6 hours; 7 hours; 8 hours; 9 hours; 10 hours	
**Circadian rythm type**			
Checkbox menu	22. Approximately what time would you get up if you were entirely free to plan your day?	5:00-6:30; 6:30-7:45; 7:45-9:45; 9:45-11:00; 11:00-12:00	Morningness-Eveningness Questionaire (MEQ)
Checkbox menu	23. Approximately what time would you go to bed if you were entirely free to plan your evening?	20:00-21:00; 21:00-22:15; 22:15-00:30; 00:30-1:45; 1:45-3:00.	Morningness-Eveningness Questionaire (MEQ)
Checkbox menu	24. If you have to get up at a specific time in the morning, how much do you depend on an alarm clock?	Not at all; Slightly; Somewhat; Very much	Morningness-Eveningness Questionaire (MEQ)
Checkbox menu	25. How easy do you find it to get up in the morning (when you are not awakened unex-pectedly)?	Very difficult; Somewhat difficult; Fairly easy; Very easy	Morningness-Eveningness Questionaire (MEQ)
Checkbox menu	26.How alert do you feel during the first half hour after you wake up in the morning?	Not at all alert; Slightly alert; Fairly alert; Very alert	Morningness-Eveningness Questionaire (MEQ)

Checkbox menu	27. How hungry do you feel during the first half hour after you wake up?	Not at all hungry; Slightly hungry; Fairly hungry; Very hungry	Morningness-Eveningness Questionaire (MEQ)
Checkbox menu	28. How tired do you feel during the first half hour after you wake up in the morning?	Very tired; Fairly tired; Fairly refreshed; Very refreshed	Morningness-Eveningness Questionaire (MEQ)
Checkbox menu	29. If you had no commitments the next day, at what time would you go to bed compared to your usual bedtime?	Seldom or never later; Less that 1 hour later; 1-2 hours later; More than 2 hours later	Morningness-Eveningness Questionaire (MEQ)
Checkbox menu	30. You have decided to engage in somephysical exercise. A friend suggests that you do this for one hour twice a week, and the best time for your friend is between 7 and 8 a.m. Bearing in mind nothing else but your own internal "clock," how do you think you would perform?	Would be in good form; Would be in reasonable form; Would find it difficult; Would find it very difficult	Morningness-Eveningness Questionaire (MEQ)
Checkbox menu	31. At approximately what time in the evening do you feel tired, and, as a result, in need of sleep?	20:00-21:00; 21:00-22:15; 22:15-00:45; 00:45-2:00; 2:00-3:00	Morningness-Eveningness Questionaire (MEQ)
Checkbox menu	32. You want to be at your peak performance for a test that you know is going to be mentally exhausting and will last two hours. You are enti-rely free to plan your day. Considering only your "internal clock," which one of the four testing times would you choose?	8:00-10:00.; 11:00-13:00; 15:00-17:00; 19:00-21:00	Morningness-Eveningness Questionaire (MEQ)
Checkbox menu	33. If you got into bed at 23:00., how tired would you be?	Not at all tired; A little tired; Fairly tired; Very tired	Morningness-Eveningness Questionaire (MEQ)
Checkbox menu	34. For some reason you have gone to bed several hours later than usual, but there is no need to get up at any particular time the next morning.	Will wake up at usual time, but will not fall back asleep; Will wake up at usual time and will doze thereafter; Will wake up at usual time, but will fall	Morningness-Eveningness Questionaire (MEQ)

	Which one of the following are you most likely to do?	asleep again; Will not wake up until later than usual	
Checkbox menu	35. One night you have to remain awake between 4:00 and 6:00 because of a night shift. You have no commitments the next day. Which one of the alternatives would suit you best?	Would not go to bed until the shift is over; Would take a nap before and sleep after; Would take a good sleep before and nap after; Would sleep only before the shift	Morningness-Eveningness Questionaire (MEQ)
Checkbox menu	36. You have two hours of hard physical work. You are entirely free to plan your day. Considering only your internal "clock," which of the following times would you choose?	8:00-10:00.; 11:00-13:00; 15:00-17:00; 19:00-21:00	Morningness-Eveningness Questionaire (MEQ)
Checkbox menu	37. You have decided to do physical exercise. A friend suggests that you do this for one hour twice a week. The best time for your friend is between 22:00-23:00. Bearing in mind only your internal "clock," how well do you think you would perform?	Would be in good form; Would be in reasonable form; Would find it difficult; Would find it very difficult	Morningness-Eveningness Questionaire (MEQ)
Checkbox menu	38. Suppose you can choose your own work hours. Assume that you work a five-hour day (including breaks), your job is interesting, and you are paid based on your performance. At approximately what time would you choose to begin?	5 hours starting between 4:00 and 8:00; 5 hours starting between 8:00 and 9:00; 5 hours starting between 9:00 and 14:00; 5 hours starting between 14:00 and 17:00; 5 hours starting between 17:00 and 4:00.	Morningness-Eveningness Questionaire (MEQ)
Checkbox menu	39. At approximately what time of day do you usually feel the best?	5:00-8:00; 8:00-10:00; 10:00-17:00; 17:00-22:00; 22:00-5:00.	Morningness-Eveningness Questionaire (MEQ)
Checkbox menu	40. One hears about "morning types" and "evening types." Which one of these types do you consider yourself to be?	Definitely a morning type; Rather more a morning type than an evening type; Rather more an evening type than a morning type; Definitely an evening type	Morningness-Eveningness Questionaire (MEQ)

Habits and life style			
Text field	41. How many units of alcohol do you drink on average per week? One unit corresponds to: 1 ordinary beer, 1 glass of wine, 1 glass of spirits (4 cl).		
Checkbox menu	42. Do you smoke (cigarettes, e-cigarettes, hookah, cigars, etc.)?	Yes, daily or almost daily; Yes, occasionally (a few times a week / at parties); No, but I once smoked daily; No, but I once smoked occasionally (a few times a week / at parties); No, I never smoked	
Checkbox menu	43. If you think about your physical activities in your spare time, inclu-ding commuting to and from work within the last year, which group do you belong to?	Almost completely physically passive or lightly physically active for less than 2 hours a week; Lightly physically active for 2-4 hours a week; Lightly physically active for more than 4 hours a week or more streneous physically active 2-4 hours a week; More strenuous physical activity for more than 4 hours or regular hard training and possibly competitions several times a week	
Checkbox menu	44. How often do you eat the following types of foods?	One or mores a day; 4-6 times a week; 3 times per week; Once or twice a week; Less than once a week; Never; Do not know	
	Fresh fruit		
	Vegetables		
	100% fruit or vegetable juice		
	Red meat (beaf, pork, lamb)		
	Processed meats (sausages and cold cuts)		

	Poultry		
	Fish		
	Eggs		
	Diary products (milk, cheese, yoghurt)		
	Pasta, rice, bread		
	Beans and lentils		
	Nuts		
	Sweets (cookies, pastries, jams, cereals with sugar)		
	Sodas with sugar		
	Fast food (fried chicken, sandwiches, pizzas, burgers)		
	Snacks (chips, crackers)		
Drop-down list	45. How many times per day do you eat fresh fruit, vegetables or drink juice?	0; 1; 2; 3; 4; 5; 6; 7; 8; 9; 10 or more	
**Health and medicine use**			
Checkbox menu	46. Have you ever had a pregnancy that lasted 24 weeks or more?	Yes; No	
Checkbox menu	47. Do you take any kind of hormones?	No; Yes, contraception e.g. IUDs, birth control pills, mini-pills; Yes, hormone therapy in connection with meno-pause; Yes, something else	
Text field	47.1 Which hormones do you take?		
Checkbox menu	48. Have you had your period in the last 12 months?	Yes; No	
Checkbox menu	48.1 What statement best describes the reason you have not had a period in the last 12 months?	Menopause; Hysterec-tomy; Ovaries removed; Currently pregnant; Currently breast feeding; Taking birth control eg.	

		hormonal IUD or hormonal contraceptives; Chemo-therapy; Hormone treat-ment; Other	
Text field	48.1.1 Suggest a reason why you have not had your period the last 12 months:		
Checkbox menu	49. Overall, how would you rate your health?	Excellent; Very good; Good; Less good; Bad	
Checkbox menu	50. Has your doctor ever told you you had any of the following conditions or diseases?		
	Myocardial infarction (blood clot in the heart)		
	Cardiac arrhythmia		
	Stroke (brain attack)		
	High cholesterol		
	Hypertension (high blood pressure)		
	Chronic bronchitis (emphysema, COPD)		
	Asthma		
	Diabetes		
	Cancer		
	Depression		
	Anxiety		
	Neurological disease (head trauma, epilepsy, dementia)		
	migraine		
Checkbox menu	51. During the past year, have you taken any of the medications listed below?	Yes; No; Don't know	
	Aspirin or other non-steroidal anti-inflamma-tory NSAID (e.g. Ibuprofen, diclofenac,		

	piroxicam) as a treatment or pain killer?		
	Medicin for heart disease or high blood pressure (e.g. beta blockers, diuretic, calcium channel blockers)		
	Medicin against depres-sion (antidepressants)		
	Melatonin supplement		
	Medicin against sleep disorder (isomnia) apart from melatonin, e.g. hypnotics		
	Cholesterol-lowering drugs (e.g. statins)		
	Diabetes medicine (metformin, insulin)		
	Other prescription drug		
	Other over-the-counter drug / natural medicine		
Checkbox menu	52. Are you taking melatonin?	No; Yes, on prescription from my own doctor; Yes, as a dietary supplement	
Checkbox menu	53. During the last four weeks, how much have you ben troubled by…	Not at all; A little troubled; Somewhat troubled; Considerably troubled; Very much troubled	
	headache?		
	neck pain?		
	pain in shoulders/arms?		
	back pain?		
	pain in hips/legs/knees/feet?		
	nausea?		
	dizziness or signs of fainting?		
	loose stools or constipation?		

	muscular pain or tension?		
	cold?		
Checkbox menu	54. How much of the time during the last two weeks…	All the time; Most of the time; Slightly more than half of the time; Slightly less than half of the time; Some of the time; Not at all	Major Depression Inventory (MDI)
	Have you felt low in spirits or sad?		Major Depression Inventory (MDI)
	Have you lost interest in your daily activities?		Major Depression Inventory (MDI)
	Have you felt lacking in energy and strength?		Major Depression Inventory (MDI)
	Have you felt less self confident?		Major Depression Inventory (MDI)
	Have you had a bad conscience or feelings of guilt?		Major Depression Inventory (MDI)
	Have you felt that life wasn't worth living?		Major Depression Inventory (MDI)
	Have you had difficulty in concentrating, e.g. when reading the newspaper or watching TV?		Major Depression Inventory (MDI)
	Have you had a reduced appetite?		Major Depression Inventory (MDI)
	Have you felt very restless?		Major Depression Inventory (MDI)
	Have you felt subdued or slowed down?		Major Depression Inventory (MDI)
	Have you had difficulties sleeping at night?		Major Depression Inventory (MDI)
	Have you had an increased appetite?		Major Depression Inventory (MDI)
**COVID-19**			
Checkbox menu	55. Have you been tested positive for COVID-19? Include self-tests and PCR-tests	Yes; No	

Checkbox menu	55.1 Have you ever been hospitalised due to COVID-19?		
Checkbox menu	55.2 How severe severely ill were you?	Hospitalized in an intensive care unit and put on a ventilator; Hospitalized in an intensive care unit (not put on a ventilator); Hospitalized, but not in an intensive care unit	
Checkbox menu	56. Have your working hours changed as a result of COVID-19, e.g. if you have been part of a medical emergency preparedness?	No; Yes, worked a lot more; Yes, worked a bit more; Yes, worked a bit less; Yes, worked a lot less	
Checkbox menu	57. Have you had other wokring hours during the COVID-19 pandemic?	No; Yes, more night work; Yes, less at night work; Yes, other	
Text field	57.1 Please describe the change in your working hours		
**Sleep - additional questions**			
	Below we will ask you further questions about your sleep		
Text fields (hour and minute)	1. During the last month, when you have not worked night shifts, when did you usually go to bed?		
Text field	2. During the past month, how long time (in minutes) has it usually taken you to fall asleep?		
Text fields (hour and minute)	3. During the last month, when did you usually get up in the morning?		
Text field	4. During the past month, how many hours did you actually sleep at night? (This might differ from the hours you spend in bed)		
Checkbox menu	5. During the past month, how often have you had trouble sleeping because you:	Not during the past month; Less than once a week; Once or twice a	

		week; Three or more times a week	
	Cannot fall asleep within 30 minutes		
	Wake up in the middle of the night or early in the morning		
	Have to get up to go to the bathroom		
	Cannot breathe comfortably		
	Cough or snore loudly		
	Feel too cold		
	Feel too hot		
	Have nightmares		
	Have pain		
	Other reasons		
Text field	5.1 Please describe other reasons why you have problems with sleeping		
Checkbox menu	6. During the past month, how would you rate your sleep quality overall?	Very good; Fairly good; Fairly bad; Very bad	
Checkbox menu	7. During the past month, how often have you taken medicine (prescribed or over-the-counter) to help you fall asleep?	Not during the past month; Less than once a week; Once or twice a week; Three or more times a week	
Checkbox menu	8. During the past month, how often have you had trouble staying awake while driving, eating meals, or engaging in social activities?	Not during the past month; Less than once a week; Once or twice a week; Three or more times a week	
Checkbox menu	9. During the past month, how much of a problem has it been for you to maintain enough enthusiasm to get things done?	Not a problem at all; Only a very slight problem; Somewhat of a problem; A very big problem	
Checkbox menu	10. During the week, how often do you take a nap?	Never; 1-2 times; 3-7 times	

Checkbox menu	10.1 How long time does the nap take on average per day?	Less than 30 minutes; 30 minutes to 1 hour; More than 1 hour	
Checkbox menu	11. How dark is your bedroom when you sleep at night?	Not dark at all (daylight); A little dark (dim); Dark (shadows are visible); Very dark (cannot see a hand extended in front of one's face)	
Checkbox menu	12. How dark is your bedroom when you sleep after a night shift?	Not dark at all (daylight); A little dark (dim); Dark (shadows visible); Very dark (cannot see a hand extended in front of one's face)	
**Health and medicine use - additional questions**			
	Below we will ask you further questions about your state of health and medicine use		
Checkbox menu	1. Over the last 2 weeks, how often have you been bothered by the following problems?	Not at all; Several days; More than half the days; Nearly every day	The generalized anxiety disorder 7-item (GAD-7) scale
	Feeling nervous, anxious or on edge		The generalized anxiety disorder 7-item (GAD-7) scale
	Not being able to stop or control worrying		The generalized anxiety disorder 7-item (GAD-7) scale
	Worrying too much about different things		The generalized anxiety disorder 7-item (GAD-7) scale
	Trouble relaxing		The generalized anxiety disorder 7-item (GAD-7) scale
	Being so restless that it is hard to sit still		The generalized anxiety disorder 7-item (GAD-7) scale
	Becoming easily annoyed or irritable		The generalized anxiety disorder 7-item (GAD-7) scale
	Feeling afraid as if something awful might happen		The generalized anxiety disorder 7-item (GAD-7) scale

Checkbox menu	2. Over the last two weeks how often have you been bothered by any of the following problems?	Not at all; Several days; More than half the days; Nearly every day	Patient Health Questionaire (PHQ-9)
	Little interest or pleasure in doing things		Patient Health Questionaire (PHQ-9)
	Feeling down, depressed, or hopeless		Patient Health Questionaire (PHQ-9)
	Trouble falling or staying asleep, or sleeping too much		Patient Health Questionaire (PHQ-9)
	Feeling tired or having little energy		Patient Health Questionaire (PHQ-9)
	Poor appetite or overeating		Patient Health Questionaire (PHQ-9)
	Feeling bad about your-self or that you are a failure or have let your-self or your family down		Patient Health Questionaire (PHQ-9)
	Trouble concentrating on things, such as reading the newspaper or watching television		Patient Health Questionaire (PHQ-9)
	Moving or speaking so slowly that other people could have noticed. Or the opposite being so fidgety or restless that you have been moving around a lot more than usual		Patient Health Questionaire (PHQ-9)
	Thoughts that you would be better off dead, or of hurting yourself		Patient Health Questionaire (PHQ-9)
Checkbox menu	2.2 If you checked off any problems, how difficult have these problems made it for you to do your work, take care of things at home, or get along with other people?	Not difficult at all; Somewhat difficult; Very difficult; Extremely difficult	Patient Health Questionaire (PHQ-9) and The genera-lized anxiety disorder 7-item (GAD-7) scale
Checkbox menu	3. In the last month, how often…	Never; Almost Never; Sometimes; Fairly Often; Very Often	Percieved Stress Scale (Cohen et al.)

	Have you been upset because of something that happened unexpectedly?		Percieved Stress Scale (Cohen et al.)
	Have you felt that you were unable to control the important things in your life?		Percieved Stress Scale (Cohen et al.)
	Have you felt nervous and "stressed"?		Percieved Stress Scale (Cohen et al.)
	Have you felt confident about your ability to handle your personal problems?		Percieved Stress Scale (Cohen et al.)
	Have you felt that things were going your way?		Percieved Stress Scale (Cohen et al.)
	Have you found that you could not cope with all the things that you had to do?		Percieved Stress Scale (Cohen et al.)
	Have you been able to control irritations in your life?		Percieved Stress Scale (Cohen et al.)
	Have you felt that you were on top of things?		Percieved Stress Scale (Cohen et al.)
	Have you been angered because of things that were outside of your control?		Percieved Stress Scale (Cohen et al.)
	Have you felt difficulties were piling up so high that you could not overcome them?		Percieved Stress Scale (Cohen et al.)
Thank you for your answers to the background questionaire. Remember to click finish below.			
