# Supplementary material for: Exposome project for health and occupational research night shift cohort (EPHOR-NIGHT): a unique resource to advance research on night shift work and chronic disease
Source: BMJ Open. 2025 Dec 5;15(12):e106090. doi: 10.1136/bmjopen-2025-106090 (PMC12684079; doi:10.1136/bmjopen-2025-106090)
Supplement: online supplemental appendix 5 [file bmjopen-15-12-s005.pdf]

EPHOR-NIGHT Ecological Momentary Application Questions and Answer Options

[note: participant needs to input their study issued ID and an app-specific password to access the application. When they do so, depending on their ID, which is linked to their study center, they will be given the appropriate language/language options]

[note: participant instructions for reminders] Please pick below what time (between 5:00-11:00) you would like to receive reminders to complete the questionnaires each day of the study

[note: they will see 2 questionnaires on their dashboard] Available questionnaires: work-sleep questionnaire, food questionnaire

[note: there are little information circles that when you click them, they say] More information, input format is HH:MM

[note: some text also that says] Thank you for answering the questionnaire, you have the following task to complete.

[note: some text also that says] Thank you for answering the questionnaire! There are no questionnaires or other tasks available at this moment.

[Note: Question 1 should be asked only on the 1st day]

1. Do you work night shifts (at least 4 hours between midnight and 06:00) or only daytime/day shifts?
    a. Permanent day only
    b. Night shift, either permanent or rotating

[Note: the following questions should be asked only 1x per day, but with 2 reminders]

2. *Reminder: please put on your heart rate monitor and use the app on your phone to start the daily heart rate recording.*

Have you put on your heart rate monitor and started the recording for today?

    a. Yes
    b. No, but I will soon
    c. I am not able to put it on today (i.e. I left it at home, etc.)

3. Did you work yesterday?
    a. Yes
    b. No, I had a day off

4. (For those responding night shift pattern to #1 and yes to #2) Did you work a night shift yesterday?
    a. Yes
    b. No

5. When did you get into bed yesterday?

    HH:MM [0/15/30/45]

6. How long did it take you to fall asleep yesterday?

    HH:MM [0/5/10/15, etc]

7. When did you wake up today?
    a. HH:MM

8. Referring to your last sleep, how would you rate your sleep quality?
    a. Terrible—excellent

9. Did you nap in the past 24 hours?
    a. Yes
    b. No

10. (For those responding yes to #8) How long of a nap did you take?
    a. HH:MM

11. Did you or will you arrive at work today?
    a. Yes
    b. I am not working today

12. (If arriving at work today in question #11 above) At what time did you or will you arrive at work today?
    a. HH:MM

13. (If arriving at work today in question #11 above) At what time did you or will you leave work today?
    a. HH:MM

14. (If arriving at work today in question #11 above) Which mode of transport did you or will you use to commute to/from work today? (multiple options allowed if your commute consisted of multiple modes of transportation)
    a. Car
    b. Bus
    c. Train
    d. Subway
    e. Tram
    f. Cycle
    g. Walk
    h. Other

15. (If arriving at work today in question #11 above) At what time did you arrive home today?

a. HH:MM

16. Did you have a caffeinated beverage (such as coffee, tea, energy drink, caffeinated soda) in the last 24 hours?
    a. Yes
    b. No

17. If yes, how many?
    a. #

18. Did you exercise in the last 24 hours (this includes vigorous activities such as heavy lifting or aerobic activity, moderate activities such as bicycling at a regular pace, and light activities such as walking and gardening/house work)?
    a. Yes
    b. No

19. What intensity level of exercise did you do? For how long did you do this exercise? And, at what time of day did you complete this activity? [note: if above answer=yes]
    a. Vigorous (vigorous physical activities include heavy lifting, digging, aerobics, fast bicycling or other activities take hard physical effort and make you breathe much harder than normal). Response in minutes
        - 17a1: What time did you complete this vigorous exercise? HH:MM *[note: if above answer>0]*

    b. Moderate (moderate activities include carrying light loads, bicycling at a regular pace, doubles tennis or other activities that take moderate physical effort and make you breathe somewhat harder than normal). Response in minutes
        - 17b1: What time did you complete this moderate exercise? HH:MM *[note: if above answer>0]*

    c. Light (Light activities require the least amount of effort such as walking slowly or light garden/house work.) Response in minutes

20. Overall in the last 24 hours, how anxious have you felt?
    a. not at all anxious --- extremely anxious

21. Overall in the last 24 hours, how depressed have you felt?
    a. not at all depressed-----most depressed

[Note: the following questions should be asked 2x per day, with 2 reminders for each]

22. Have you had any meals or snacks in the last 12 hours?
    a. Yes
    b. No

23. (if “yes” to question above) Was this a snack or a main meal?
    a. Snack
    b. Breakfast
    c. Lunch
    d. Dinner

24. What time did you eat this snack or meal?
    a. HH:MM

25. Please indicate the category or categories of food you ate (multiple responses allowed):
    a. Fruits (juice is not included)
    b. Vegetables- non-starchy (e.g. asparagus, cucumber, broccoli, pepper, etc., leafy greens, etc.)
    c. Vegetables- starchy (e.g. potatoes, sweet potato, squash, etc.)
    d. Whole grains (e.g. brown rice, whole wheat bread or pasta, oatmeal)
    e. Refined grains (white rice, pasta, bread, refined breakfast cereals)
    f. Nuts, seeds or legumes (includes products that are based from legumes/nuts and seeds like tofu, tempeh, tahini and vegan alternatives)
    g. Dairy (e.g. cheese, butter, milk, cream, yogurt)
    h. Fish
    i. Red meat (e.g. from cow, lamb or pig)
    j. Processed meat (e.g. salami, sausage, salted and cured meats)
    k. Poultry (e.g. chicken, turkey, etc. (not red)) and eggs
    l. “Fast food” or pre-prepared food (e.g. burgers, fried chicken pieces, French fries)
    m. Sugar-sweetened beverages (e.g. soda, sweetened tea, commercial juice)
    n. Cookies/pastries/candy
    o. Other
    p. Alcoholic drinks (wine, beer, spirit, other)
    q. Coffee or tea

26. Have you had any other meals or snacks in the last 12 hours?
    a. Yes *[note: if yes, they will be shown questions 22-25 again until they have been able to enter in all of their meals/snacks in the relevant time period]*
    b. No
