# Supplementary material for: Exposome project for health and occupational research night shift cohort (EPHOR-NIGHT): a unique resource to advance research on night shift work and chronic disease
Source: BMJ Open. 2025 Dec 5;15(12):e106090. doi: 10.1136/bmjopen-2025-106090 (PMC12684079; doi:10.1136/bmjopen-2025-106090)
Supplement: online supplemental appendix 6 [file bmjopen-15-12-s006.pdf]

National Research Centre for the Working Environment

Sleep diary questionaire, English

Thank you for participating in the research project "1001 nights". The sleep diary is completed once every day - when you wake up from your primary sleep. We will ask you questions regarding your sleep, work, diet and exercise during the last 24 hours. Among other things, we will ask you to indicate when you were on a shift and when you slept.*

Time is stated in hours and minutes.
Examples: 11.30 p.m. is referred to as:
HOUR: 23 MINUTE: 30
7 a.m. is referred to as:
HOUR: 07 MINUTE: 00
**) For the remaining sleep diaries the reference period was "since filling in the previous sleep diary" which could cover fewer or more than 24 hours.*

Choice of date in calender (dd-mm-yyyy)	1. Please indicate the date
	*In the case of (typing) errors, the participants got the following message: 'Please note that **the indicated date is different from todays date**.*<br>*If that is correct, continue by clicking "next". If You would like to correct the date, click "previous".*<br>*The sleep diary should preferably be filled in the day you recieved it. Consequently, the date should agree with the day you recieved the diary.*<br>*Have you forgotten a fill out a sleep diary?*<br>*If you have forgotten to fill out a sleep diary, you can complete it at a later date. Please make sure to fill out the right sleep diary - that is, the sleep diary you recived on the day, where you forgot to fill out the diary. You should indicate the date, when the diary were intended to be filled out - that is, the day you recieved the sleep diary.*<br>*Example: Tuesday morning you recieved sleep diary 1 and you forgot to complete it. On Wednesday, you wish to fill out the forgotten diary, however, you have two links in your inbox: Diary 1 and diary 2.*<br>*Solution: To fill in the sleep diary you forgot on Tuesday, open diary 1 (and not diary 2). Indicate the date for Tuesday - even though you fill in the diary on Wednesday. Also fill in the sleep diary of the day (diary 2) and indicate the date for Wednesday.*
	*In the case of (typing) errors, the participants got the following message: **You indicated a date, which is later than todays date.***<br>*Click "previous" to correct the date.*<br>*The sleep diary should preferably be filled in the day you recieved it. Consequently, the date should agree with the day you recieved the diary.*

Type of answer to the question	Component	Response options if applicable	Documentation
Checkbox menu	1.1. Did you wear your Actigraph during your primary sleep?	Yes; No; I haven't had a primary sleep period du-ring the last 24 hours (do not count in naps)	
Text fields (hour and minutes)	2. When did you go to bed?		
Text field	3. How long did it take you to fall asleep? (indicate minutes)		
Text fields (hour and minutes)	4. When did you wake up?		
Checkbox menu	5. Was it difficult to fall asleep?	Not at all; A bit; Somewhat difficult; Quite difficult; Very difficult	
Checkbox menu	6. How would you decribe your sleep?	Very good; Fairly good; Neither good nor poor; Fairly poor; Very poor	
Checkbox menu	7. Were you restless in your sleep?	Not at all; A bit; Somewhat restless; Quite restless; Very restless	
Checkbox menu	8. Did you wake up too early, unable to fall asleep again?	No; Yes, sligthly too early; Yes, very early	
Checkbox menu	9. How many times did you wake up during your sleep?	0 times; once; twice; three times; four times or more	
Checkbox menu	10. How easy was it to get out of bed?	Very easy; Easy; Neither easy nor difficult; Difficult, Very difficult	
Checkbox menu	11. How rested do you feel?	Completely rested; Very rested; Quite rested; Little rested; Not at all rested	
Checkbox menu	12. Did you drink alcohol or take medicine before you went to bed? (You may choose more than one response option)	No; Yes, sleeping medicine; Yes, alcohol; Yes, analgesic; Ja, melatonin; Yes, something else	
Text field	12.1 Please describe "something else"		

**Work**			
Checkbox menu	13. Did you work during the last 24 hours?	Yes; No	
Text fields (hour and minute)	13.1 When did your shift start?		
Text fields (hour and minute)	13.2 When did your shift end?		
If you had more than one shift during the last 24 hours, please indicate when your second shift started and ended.			
Text fields (hour and minute)	13.3 When did your second shift start?		
Text fields (hour and minute)	13.4 When did your second shift end?		
Numeric scale	13.5 How busy was your shift/your shifts? Choose on a scale from 1 through 10. 1 means "not busy" and 10 means "extremely busy"	1 Not busy; 2; 3; 4; 5; 6; 7; 8; 9; 10 Extremely busy	
Numeric scale	13.6 How physically demanding was your shift/your shifts?	1 Not demanding; 2; 3; 4; 5; 6; 7; 8; 9; 10 Extremely demanding	
Numeric scale	13.7 How emotionally demanding was your shift/ your shifts?	1 Not demanding; 2; 3; 4; 5; 6; 7; 8; 9; 10 Extremely demanding	
	How much do you agree with the following statements?	1 Disagree; 2; 3; 4; 5 Agree	
	13.8 I was exhausted by the end of my shift.	1 Disagree; 2; 3; 4; 5 Agree	
	13.9 Right after retur-ning home from work, it was difficult to show interest in other people.	1 Disagree; 2; 3; 4; 5 Agree	
**Naps**			
Checkbox menu	14. During the last 24 hours, have you taken a nap/powernap one or more times?	Yes; No	
Text fields (hour and minute)	14.1 For up to four naps, please indicate when you fell asleep and when you woke up		

	1st nap: Please state when you fell asleep		
	1st nap: Please state when you woke up		
	2nd nap: Please state when you fell asleep		
	2nd nap: Please state when you woke up		
	3rd nap: Please state when you fell asleep		
	3rd nap: Please state when you woke up		
	4th nap: Please state when you fell asleep		
	4th nap: Please state when you woke up		
**Meals**			
	Below, we wil ask you to register your meals during the last 24 hours.<br>*A main meal is a larger meal, eg. breakfast, lunch or dinner.*<br>*A snack might be a granola bar, a piece of fruit, a piece of bread or a similar meal.*		
Drop-down list	15. During the last 24 hours, when did you eat a main meal? Please indicate up to four main meals.	Every hour and half-hour starting at 00:00.	
	1st main meal		
	2nd main meal		
	3rd main meal		
	4th main meal		
Drop-down list	15.1. During the last 24 hours, when did you eat a snack? Please indicate up to four snacks.		
	1st snack		
	2nd snack		
	3rd snack		
	4th snack		

Physical activity			
	Below we will ask questions about physical activity.		
Checkbox menu	16.1 During the last 24 hours, did you do light intensity activites?	Yes, light intensity actvities that required little effort, eg. a slow walk, gardening or housework.; No	
Drop-down list	16.1.1 Please state total time in minutes spent with light intensity activity during the last 24 hours	0-10 minutes;<br>11-20 minutes;<br>21-30 minutes;<br>31-40 minutes;<br>41-50 minutes;<br>51-60 minutes;<br>61-70 minutes;<br>71-80 minutes;<br>81-90 minutes;<br>91-100 minutes;<br>101-110 minutes; 111-120 minutes;<br>More than 120 minutes (2 hours)	
Text fields (hour and minute)	16.1.2 Please state when you did light intensity activity. If you have had several periods of light intensity activity, please indicate starting time for up to 4 periods of light intensity activity.		
Text fields (hour and minute)	Please state the starting time for your second period of ligth intensity activity		
Text fields (hour and minute)	Please state the starting time for your third period of ligth intensity activity		
Text fields (hour and minute)	Please state the starting time for your fourth period of ligth intensity activity		
Checkbox menu	16.2 During the last 24 hours, did you do moderate intensity activites?	Yes, moderate intensity activity: Activities demanding a moderate effort and which causes small increases in breathing, eg. brisk	

		walking or light intensity biking.; No	
Drop-down list	16.2.1 Please state total time in minutes spent with moderate intensity activity during the last 24 hours	0-10 minutes;<br>11-20 minutes;<br>21-30 minutes;<br>31-40 minutes;<br>41-50 minutes;<br>51-60 minutes;<br>61-70 minutes;<br>71-80 minutes;<br>81-90 minutes;<br>91-100 minutes;<br>101-110 minutes;<br>111-120 minutes;<br>More than 120 minutes (2 hours)	
Text fields (hour and minute)	16.2.2 Please state when you did moderate intensity activity. If you have had several periods of moderate intensity activity, please indicate starting time for up to 4 periods of moderate intensity activity.		
Text fields (hour and minute)	Please state staring time for your second period of moderate intensity activity		
Text fields (hour and minute)	Please state staring time for your third period of moderate intensity activity		
Text fields (hour and minute)	Please state staring time for your fourth period of moderate intensity activity		
Checkbox menu	16.3 During the last 24 hours, did you do vigorous-intensity activities?	Yes, vigorous-intensity activity: Activities demanding great effort and cause you to breathe heavily, eg. running or heavy lifts.; No	
Drop-down list	16.3.1 Please state total time in minutes spent with vigorous-intensity activity during the last 24 hours	0-10 minutes; 11-20 minutes; 21-30 minutes; 31-40 minutes; 41-50 minutes; 51-60 minutes; 61-70 minutes; 71-80	

		minutes; 81-90 minutes; 91-100 minutes; 101-110 minutes; 111-120 minutes; More than 120 minutes (2 hours)	
Text fields (hour and minute)	16.3.2 Please state when you did vigorous-intensity activity. If you have had several periods of vigorous-intensity activity, please indicate starting time for up to 4 periods of vigorous-intensity activity.		
Text fields (hour and minute)	16.3.2 Please state staring time for your second period of vigorous-intensity activity		
Text fields (hour and minute)	16.3.2 Please state staring time for your third period of vigorous-intensity activity		
Text fields (hour and minute)	16.3.2 Please state staring time for your fourth period of vigorous-intensity activity		
**Health**			
Checkbox menu	17. During the last 24 hours, have you experienced any of the symptoms listed below? (You may choose more than one option).	No symptoms; Headache; Pain in the neck; Pain in the shoulders/ arms; Backpain; Pain in the hips/legs/knees/feet; Other symptoms	
Text field	17.1 Please describe "other symptoms"		
Thank you for your answers!			
