# Supplementary material for: Exposome project for health and occupational research night shift cohort (EPHOR-NIGHT): a unique resource to advance research on night shift work and chronic disease
Source: BMJ Open. 2025 Dec 5;15(12):e106090. doi: 10.1136/bmjopen-2025-106090 (PMC12684079; doi:10.1136/bmjopen-2025-106090)
Supplement: online supplemental appendix 7 [file bmjopen-15-12-s007.docx]

**Supplemental Appendix 7:** **Demographic characteristics of the EPHOR-NIGHT sample, further stratifying the Spanish center by health versus transportation sector (N=937)^a^**

| Characteristics | Spain-Healthcare n=370 | Spain-Transport n=33 | Sweden n=170 | Denmark n=294 | the Netherlands n=70 |
| --- | --- | --- | --- | --- | --- |
| Age (mean (SD)) | 42.54 (13.58) | 46.38 (8.51) | 46.70 (10.80) | 42.03 (11.85) | 51.42 (9.35) |
| Female | 306 (82.7) | <15%* | 151 (88.8) | 294 (100.0) | 70 (100.0) |
| European origin | 321 (86.8) | 31 (96.9) | 132 (77.6) | 282 (95.9) | - |
| Education |  |  |  |  |  |
| Secondary studies or less | 150 (40.5) | 27 (81.8) | 45 (26.5) | 53 (18.0) | 19 (27.1) |
| University or more | 220 (59.5) | 6 (18.2) | 125 (73.5) | 241 (82.0) | 51 (72.9) |
| Current smokers | 102 (27.6) | <15%* | 38 (22.4) | 49 (16.7) | 2 (2.9) |
| Current alcohol use | 224 (60.5) | 23 (69.7) | 147 (86.5) | - | 53 (75.7) |
| Marital status |  |  |  |  |  |
| Married or living as a couple | 194 (52.4) | 24 (72.7) | 111 (65.3) | 218 (74.1) | 51 (72.9) |
| In relationship but living alone | 34 (9.2) | <15%* | 12 (7.1) | 27 (9.2) | 5 (7.1) |
| Single | 96 (26.0) | <15%* | 33 (19.4) | 36 (12.2) | 6 (8.6) |
| Other | 46 (12.4) | 5 (15.1) | 14 (8.2) | 13 (4.4) | 8 (11.4) |
| Night shift worker | 194 (52.4) | 17 (51.5) | 85 (50.0) | 206 (70.1) | 62 (88.6) |
| Have previously worked night shifts | 279 (75.4) | 30 (90.9) | 66 (64.7) | 232 (81.1) | 69 (100.0) |
| Existing conditions^b^ |  |  |  |  |  |
| Cardiac arrhythmia | 19 (5.1) | <15%* | 14 (8.3) | 9 (3.1) | 8 (11.8) |
| High cholesterol/triglycerides | 99 (26.8) | 14 (43.8) | 7 (4.2) | 38 (12.9) | 8 (11.9) |
| Hypertension | 37 (10.0) | 7 (21.9) | 29 (17.2) | 28 (9.5) | 7 (10.1) |
| Asthma | 47 (12.7) | <15%* | 32 (19.0) | 35 (11.9) | 1 (1.5) |
| Depression | 71 (19.2) | <15%* | 46 (27.4) | 43 (14.6) | 5 (7.4) |
| Anxiety | 139 (37.6) | 10 (31.2) | 52 (31.0) | 33 (11.2) | - |

*Due to privacy and ethical concerns, when the membership in a given cell was fewer than 5 individuals (<15% of the population), we have indicated <15%

Missing values: % are calculated from non-missing values. Data were missing for alcohol use 31%, all other variables <1%

“-“ empty cells indicate that these questions were not asked or relevant

^a^ n (%) for categorical variables and mean (SD) for continuous variables

^b^ In Spain, Sweden and Denmark this question was worded, “Has your doctor ever told you that you have any of the following diseases…?” While in The Netherlands it was worded, “Has your doctor told you after January 1^st^, 2017 that you have…?”

Abbreviations: SD: standard deviation
