# Supplementary material for: Exposome project for health and occupational research night shift cohort (EPHOR-NIGHT): a unique resource to advance research on night shift work and chronic disease
Source: BMJ Open. 2025 Dec 5;15(12):e106090. doi: 10.1136/bmjopen-2025-106090 (PMC12684079; doi:10.1136/bmjopen-2025-106090)
Supplement: online supplemental appendix 8 [file bmjopen-15-12-s008.docx]

**EPHOR Night Study Consortium**

**Analysis Concept Form**

**to analyse EPHOR-NIGHT data**

Please provide the following information on the analyses you wish to perform and send via email to Gemma Castaño [gemma.castano@isglobal.org](mailto:gemma.castano@isglobal.org). You can also contact any of the other national PIs of the study: Karin Broberg [karin.broberg@med.lu.se](mailto:karin.broberg@med.lu.se) for Sweden; 'Anne Helene Garde' [AHG@nfa.dk](mailto:AHG@nfa.dk) for Denmark; Susan Peters [S.Peters@uu.nl](mailto:S.Peters@uu.nl) for The Netherlands; and Barbara N Harding [BNHarding@salud.unm.edu](mailto:BNHarding@salud.unm.edu) for Spain.

The form will be reviewed by the EPHOR-NIGHT steering committee which will help to identify overlaps, suggest potential merges and organize proposals so that they can be presented to the PIs. ***The final decisions with regard to each analysis, and who participates, rest with the study PIs.***

| **Date**: |  |
| --- | --- |

| **Title of Proposed Project**: |  |
| --- | --- |

| **Investigators:** |  |
| --- | --- |
| **Investigator Institutions:** | . |
| **Contact e-mail** |  |

| **Concept Description**: *Please provide a concise description of Background/Aims* |
| --- |
| **Keywords:** *Please provide a few words indicating the topic area of the proposal* |

| **Primary Endpoint**:   \|  \| *Yes or No* \|  \| \| --- \| --- \| --- \| \| Mental health- Cognition \|  \|  \| \| Cardiovascular \|  \|  \| \| Sleep \|  \|  \| \| Other \|  \| Specify: \| |
| --- | --- | --- | --- | --- | --- | --- | --- | --- | --- | --- | --- | --- | --- | --- | --- |

| **Biomarker/OMICS data**: *Please indicate the biomarker/OMICS data that is being requested:*   \|  \| *Yes or no* \| *Specify (if necessary)* \| \| --- \| --- \| --- \| \| GWAS data \|  \|  \| \| Metabolomics \|  \|  \| \| Proteomics \|  \|  \| \| Inflammatory Markers \|  \|  \| \| Hormones (melatonin, cortisol, sex-steroid) \|  \|  \| \| Microbiome \|  \|  \| \| EWAS-epigenetics \|  \|  \| \| Targeted DNA Methylation \|  \|  \| \| Telomere length and mtDNA copy number \|  \|  \| \| Metals \|  \|  \| |
| --- | --- | --- | --- | --- | --- | --- | --- | --- | --- | --- | --- | --- | --- | --- | --- | --- | --- | --- | --- | --- | --- | --- | --- | --- | --- | --- | --- | --- | --- | --- | --- | --- | --- |
| **SNPs**: *If necessary please provide in an attached Excel file the specific list biomarkers/omics (e.g. SNPs) requested. Please indicate the reason for the request of these data* |

| **Sensors**: *Sensors may not be available from all the studies.*   \|  \| *Yes or No* \| *Specify (if necessary)* \| \| --- \| --- \| --- \| \| HOBO Light Intensity Logger \|  \|  \| \| Kronowise Wrist Device \|  \|  \| \| Heart Rate Monitor (Polar H10 \|  \|  \| \| Other \|  \| Specify: \| |
| --- | --- | --- | --- | --- | --- | --- | --- | --- | --- | --- | --- | --- | --- | --- | --- |

| **Core Variables**: *These are the most common variables in the EPHOR-NIGHT consortium although they may not be available from all the studies. Please indicate the request of variables*   \|  \|  \| *Yes or No* \| *Notes* \| \| --- \| --- \| --- \| --- \| \| **Baseline questionnaire** \| lifestyle \|  \|  \| \| medical history \|  \|  \| \| sleep \|  \|  \| \| diet \|  \|  \| \| working conditions \|  \|  \| \| night-shift duration \|  \|  \| \| psychosocial factors \|  \|  \| \| **Ecological momentary assessment** \|  \|  \|  \| \| **Anthropomorphic /Clinical measurements** \| Height \|  \|  \| \| Weight \|  \|  \| \| Waste Hip \|  \|  \| \| Blood perssure \|  \|  \| \| Other \|  \|  \| |
| --- | --- | --- | --- | --- | --- | --- | --- | --- | --- | --- | --- | --- | --- | --- | --- | --- | --- | --- | --- | --- | --- | --- | --- | --- | --- | --- | --- | --- | --- | --- | --- | --- | --- | --- | --- | --- | --- | --- | --- | --- | --- | --- | --- | --- | --- | --- |
| **Other variables**: *Please check the EPHOR-NIGHT data inventory for other variables than the above indicated core ones. Note those variables may not be available for all the studies in the consortium. Provide below the list of other variables that are requested for your analysis.* |

| **Additional Requests:** *For studies requiring biological specimens, please specify sample request and sample requirements.* *Please also provide information on methods/assays, if applicable (e.g. specification of antibody and probes and method of scoring).* |
| --- |

| **Analysis Plan:** *Please include power considerations*. |
| --- |

| **Budgetary considerations, if applicable:** |
| --- |

| **Writing committee and authorship agreements:**  NB any data using EPHOR-NIGHT data and/or samples should comply with the EPHOR-NIGHT authorship guidelines*:*   - *For papers using the whole dataset* at least 2 authors per data study used and list of all other authors relevant under “and the EPHOR-NIGHT consortium”. This list could include extra members in a group who has had a contribution and could be in foot notes or on the supplementary material. - *For papers using a limited number of data/biomarkers* 1 author per data study used and list of all other authors relevant under “and the EPHOR-NIGHT consortium”.   <indicate writing committee> |
| --- |

| **Time line:** |
| --- |
