# Supplementary material for: Exposome project for health and occupational research night shift cohort (EPHOR-NIGHT): a unique resource to advance research on night shift work and chronic disease
Source: BMJ Open. 2025 Dec 5;15(12):e106090. doi: 10.1136/bmjopen-2025-106090 (PMC12684079; doi:10.1136/bmjopen-2025-106090)
Supplement: Supplementary file 9 [file bmjopen-2025-106090.reviewer_comments.pdf]

PEER REVIEW HISTORY

BMJ Open publishes all reviews undertaken for accepted manuscripts. Reviewers are asked to complete a checklist review form and are provided with free text boxes to elaborate on their assessment. These free text comments are reproduced below.

ARTICLE DETAILS

Title (Provisional)

Cohort profile: The Exposome Project for Health and Occupational Research Night Shift Cohort (EPHOR-NIGHT), a unique resource to advance research on night shift work and chronic disease

Authors

Harding, Barbara; Castaño-Vinyals, Gemma; Broberg, Karin; Albin, Maria; Laurell, Caisa; Garde, Anne Helene; Nabe-Nielsen, Kirsten; Fiehn, Anne Emily Saunte; van der Grinten, Tara; Peters, Susan; Vermeulen, Roel C.H.; Gosh, Manosij; Loh, Miranda; Pronk, Anjoeka; Kogevinas, Manolis

---

VERSION 1 - REVIEW

**Reviewer**	**1**
**Name**	**Benavides, Fernando**
**Affiliation**	**Center for Research in Occupational Health - Pompeu Fabra University**
**Date**	**17-Sep-2025**
**COI**	**None**

The study addresses a highly relevant topic, and the research program's long-term goal of developing preventive strategies is commendable. The paper is well-written, but I have a few major and minor comments that I believe would strengthen the manuscript.

The theoretical model for this study is complex, involving multiple direct and indirect pathways, more of then bidirectional, between night work, lifestyle, and health outcomes like cancer and other non-communicable diseases. To help readers better understand these intricate relationships, I strongly recommend including a causal diagram at the end of the Introduction section. This diagram would visually clarify the core hypotheses and the different mechanisms being explored within the exposome framework.

While the paper's focus on the biological mechanism is a key part of the EPHOR-NIGHT cohort, it is not sufficient to achieve the stated goal of identifying preventive strategies. A comprehensive understanding of health effects requires a consideration of the social and organizational context of work. I suggest the authors to expand the questionnaire to include more variables on working conditions. Incorporating items from a reference tool like the last (2024) European Working Conditions Survey (e.g., questions on the physical and social environment, work intensity, and income) would be a valuable addition. This would allow the study to explore the crucial interaction between social and biological mechanisms.

The current cohort size appears to be a major limitation for a broad, multifactorial analysis. The current size seems most suitable for generating verifiable knowledge about the biological pathway between night work and the circadian cycle. This focus would ensure that the study's conclusions are robust and well-supported by the available data.

However, more detailed information is needed on the ongoing follow-up activities at the Spanish and Swedish centers. Clearly describing these activities would not only give the cohort study a more robust longitudinal character but also reinforce the potential to incorporate new data on occupational and work conditions, as well as biological analyses (e.g., microbiome and epigenetic profiles).

In the case of Spain sample, to provide a more accurate and useful comparison, it would be best to separate the workers from TMB and those from Hospital del Mar, as they represent very different sectors and employment models.

From a prevention standpoint, it is essential to include a reference to Directive 2003/88/EC of the European Parliament and of the Council concerning certain aspects of the organization of working time. This directive provides a foundational and legally-grounded definition of night work (at least three hours of the working day during the night), which would be a useful basic definition for the study.

VERSION 1 - AUTHOR RESPONSE

Reviewer: 1
Comments to the Authors:
The study addresses a highly relevant topic, and the research program's long-term goal of developing preventive strategies is commendable. The paper is well-written, but I have a few major and minor comments that I believe would strengthen the manuscript.

The theoretical model for this study is complex, involving multiple direct and indirect pathways, more of then bidirectional, between night work, lifestyle, and health outcomes like cancer and other

non-communicable diseases. To help readers better understand these intricate relationships, I strongly recommend including a causal diagram at the end of the Introduction section. This diagram would visually clarify the core hypotheses and the different mechanisms being explored within the exposome framework.

Response: Thank you for this suggestion. We have included a model (Figure 1) to serve as an overview of the core hypotheses and various mechanisms and outcomes of interest. The figure is mentioned in the text (lines 143-144), and is included below for reference.

Figure 1. Conceptual framework of the EPHOR-NIGHT cohort showing the exposome approach to studying night shift work and health. Night shift and related occupational, behavioural, and environmental exposures lead to circadian, metabolic, immune, and molecular changes that are associated with mental, cognitive, and cardiometabolic outcomes, modulated by chronotype, genetic variation, sex, and sleep quality.

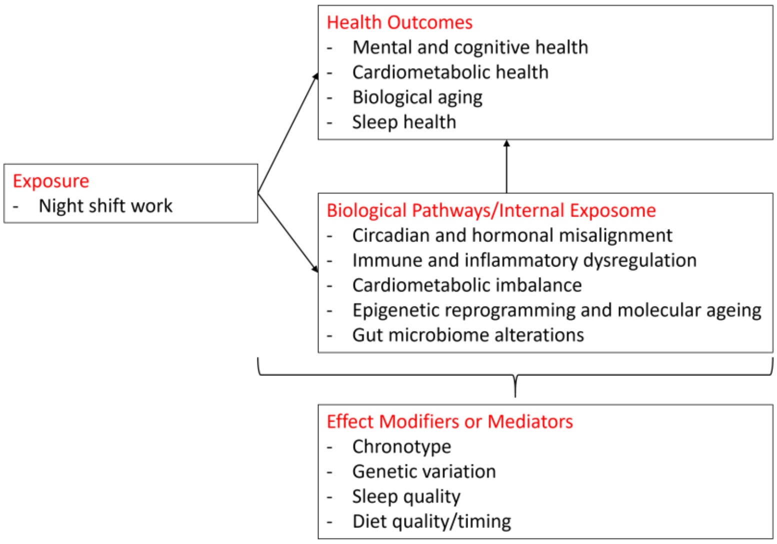

While the paper's focus on the biological mechanism is a key part of the EPHOR-NIGHT cohort, it is not sufficient to achieve the stated goal of identifying preventive strategies. A comprehensive understanding of health effects requires a consideration of the social and organizational context of work. I suggest the authors to expand the questionnaire to include more variables on working conditions. Incorporating items from a reference tool like the last (2024) European Working Conditions Survey (e.g., questions on the physical and social environment, work intensity, and income) would be a valuable addition. This would allow the study to explore the crucial interaction between social and biological mechanisms

Response: We have completed the data collection process for the cohort, as well as a 2-year follow up effort for participants in the Spanish and Swedish cohort. As these phases of the data collection process have completed, we cannot make modifications to add additional questions to the questionnaires. However, we did include several questions in the follow-up survey on working conditions using the COPSOQ questionnaire which collects information on qualitative demands, workplace, emotional demands, control, social support, etc. We are working to clean these data for their use in future analyses.

The current cohort size appears to be a major limitation for a broad, multifactorial analysis. The current size seems most suitable for generating verifiable knowledge about the biological pathway

between night work and the circadian cycle. This focus would ensure that the study's conclusions are robust and well-supported by the available data.

Response: A major limitation highlighted by various expert committees evaluating the effects of night shift work on health, for example in the IARC 2019 Monograph, was the lack of large and well-conducted mechanistic studies in human populations. We agree that with our study, which is probably the largest and most complete to date, we will be able to provide solid evidence on specific biological pathways linking night shift work and circadian disruption. While we are planning to conduct multifactorial and multi-omics analyses, we acknowledge that the power for some of these analyses will be limited. Nonetheless, we consider that these analyses remain valuable, given the uniqueness of the dataset and the absence of other population-based studies with comparable depth and harmonization across multiple countries.

We have added text to the discussion section (lines 409-412) related to this:
"While the EPHOR-NIGHT study represents one of the largest and most integrative mechanistic investigations of night shift work in human populations to date, which will allow for the identification of specific biological pathways underlying circadian disruption, the cohort size may limit statistical power for highly multifactorial or multi-omics analyses."

However, more detailed information is needed on the ongoing follow-up activities at the Spanish and Swedish centers. Clearly describing these activities would not only give the cohort study a more robust longitudinal character but also reinforce the potential to incorporate new data on occupational and work conditions, as well as biological analyses (e.g., microbiome and epigenetic profiles).

Response: We have expanded the "future plans" section in the abstract (lines 58-61) which now reads, A two-year follow-up is ongoing, with planned integration of microbiome and further omics analyses, as well as new questions on psychosocial work environment and data related to female sexual and reproductive health. Findings aim to inform prevention strategies and occupational health policies. Data will be made available to support broader research efforts on shift work and health."
We have also expanded the "future plans" section in the main manuscript (lines 417-429) to add some additional aspects covered in the follow-up efforts.

Per the editor guidelines above, the purpose of the profile paper is not to provide data on the findings from follow-up analyses. These forthcoming results will be published in future manuscripts. For the time-being, we wanted to provide an overview of the creation of the cohort and the data types available from the participants.

In the case of Spain sample, to provide a more accurate and useful comparison, it would be best to separate the workers from TMB and those from Hospital del Mar, as they represent very different sectors and employment models.

Response: Thank you for this comment. We agree that the transportation workers preferably should be presented separately. They are however a very small group (n=33), as we had difficulties recruiting participants outside of the health system; mainly due to complex protocols and the less fixed shift schedules in this sector as compared to the healthcare sector. We have added a supplemental table that further breaks down the Spanish group into transport versus healthcare workers. We mention this new supplemental table in the manuscript text (lines 363-369), where we write: "In Supplemental Appendix 7 we further stratify the Spanish sample into transportation workers (n=33) and healthcare workers (n=370) to describe demographic differences between

workers in each of these sectors, finding that the transportation workers were more often men, more likely to have completed secondary studies or less, more likely to be married or living as a couple, more likely to have previously worked night shifts, reported lower levels of smoking but higher levels of current alcohol use and had a different distribution of existing conditions than those in the healthcare sector."

The new supplemental table is included below for reference:

Supplemental Appendix 7: Demographic characteristics of the EPHOR-NIGHT sample, further stratifying the Spanish center by health versus transportation sector (N=937)[a]

Characteristics	Spain-Healthcare n=370	Spain-Transport n=33	Sweden n=170	Denmark n=294	the Netherlands n=70
Age (mean (SD))	42.54 (13.58)	46.38 (8.51)	46.70 (10.80)	42.03 (11.85)	51.42 (9.35)
Female	306 (82.7)	<15%*	151 (88.8)	294 (100.0)	70 (100.0)
European origin	321 (86.8)	31 (96.9)	132 (77.6)	282 (95.9)	-
Education					
Secondary studies or less	150 (40.5)	27 (81.8)	45 (26.5)	53 (18.0)	19 (27.1)
University or more	220 (59.5)	6 (18.2)	125 (73.5)	241 (82.0)	51 (72.9)
Current smokers	102 (27.6)	<15%*	38 (22.4)	49 (16.7)	2 (2.9)
Current alcohol use	224 (60.5)	23 (69.7)	147 (86.5)	-	53 (75.7)
Marital status					
Married or living as a couple	194 (52.4)	24 (72.7)	111 (65.3)	218 (74.1)	51 (72.9)
In relationship but living alone	34 (9.2)	<15%*	12 (7.1)	27 (9.2)	5 (7.1)
Single	96 (26.0)	<15%*	33 (19.4)	36 (12.2)	6 (8.6)
Other	46 (12.4)	5 (15.1)	14 (8.2)	13 (4.4)	8 (11.4)
Night shift worker	194 (52.4)	17 (51.5)	85 (50.0)	206 (70.1)	62 (88.6)
Have previously worked night shifts	279 (75.4)	30 (90.9)	66 (64.7)	232 (81.1)	69 (100.0)
Existing conditions[b]					
Cardiac arrhythmia	19 (5.1)	<15%*	14 (8.3)	9 (3.1)	8 (11.8)
High cholesterol/triglycerides	99 (26.8)	14 (43.8)	7 (4.2)	38 (12.9)	8 (11.9)
Hypertension	37 (10.0)	7 (21.9)	29 (17.2)	28 (9.5)	7 (10.1)
Asthma	47 (12.7)	<15%*	32 (19.0)	35 (11.9)	1 (1.5)
Depression	71 (19.2)	<15%*	46 (27.4)	43 (14.6)	5 (7.4)
Anxiety	139 (37.6)	10 (31.2)	52 (31.0)	33 (11.2)	-

*Due to privacy and ethical concerns, when the membership in a given cell was fewer than 5 individuals (<15% of the population), we have indicated <15%

Missing values: % are calculated from non-missing values. Data were missing for alcohol use 31%, all other variables <1%

"-" empty cells indicate that these questions were not asked or relevant

[a] n (%) for categorical variables and mean (SD) for continuous variables

[b] In Spain, Sweden and Denmark this question was worded, "Has your doctor ever told you that you have any of the following diseases…?" While in The Netherlands it was worded, "Has your doctor told you after January 1st, 2017 that you have…?"

Abbreviations: SD: standard deviation

From a prevention standpoint, it is essential to include a reference to Directive 2003/88/EC of the European Parliament and of the Council concerning certain aspects of the organization of working time. This directive provides a foundational and legally-grounded definition of night work (at least three hours of the working day during the night), which would be a useful basic definition for the study.

Response: We agree that we should mention Directive 2003/88/EC. In the EPHOR-NIGHT study, night-shift workers were defined according to the study protocol as those working a minimum of two consecutive night shifts per week and having at least three years of night-shift history prior to enrolment. A night shift was defined as any working schedule involving ≥4 hours between 00:00 and 06:00, following a slightly modified version of the International Agency for Research on Cancer (IARC) consensus definition for night-shift work. This definition closely aligns with the European Directive 2003/88/EC, which defines night work as including at least three hours between 00:00 and 05:00. While the Directive provides the legal framework for regulating working time in the EU, our operational definition was selected to ensure comparability with prior epidemiological studies and to capture a broader range of biologically relevant night-time exposure.

To the discussion we have added (lines 430-438): "From a prevention and regulatory perspective, our findings are directly relevant to the European Directive 2003/88/EC, which defines night work as employment involving at least three hours of the working day between 00:00 and 05:00, a definition that is closely aligned with our night shift definition of any working schedule involving ≥4 hours between 00:00 and 06:00. While the Directive provides the legal framework for regulating working time in the EU, our operational definition was selected to ensure comparability with prior epidemiological studies and to capture a broader range of biologically relevant night-time exposure. Incorporating such definitions into research studies helps align epidemiological research with occupational health policy and supports evidence-based recommendations for safer scheduling practices."

*** ***

COI statement(s):

Reviewer: 1
If you have selected 'Yes' above, please provide details of any competing interests.: Not applicable.
